# Improving Deep Interactive Evolution with a Style-Based Generator for Artistic Expression and Creative Exploration

**DOI:** 10.3390/e23010011

**Published:** 2020-12-24

**Authors:** Carlos Tejeda-Ocampo, Armando López-Cuevas, Hugo Terashima-Marin

**Affiliations:** School of Engineering and Sciences, Tecnologico de Monterrey, 64849 Monterrey, Mexico; acuevas@tec.mx (A.L.-C.); terashima@tec.mx (H.T.-M.)

**Keywords:** generative adversarial networks, interactive evolutionary computation, deep interactive evolution, StyleGAN, latent space exploration, neural art, evolutionary art

## Abstract

Deep interactive evolution (DeepIE) combines the capacity of interactive evolutionary computation (IEC) to capture a user’s preference with the domain-specific robustness of a trained generative adversarial network (GAN) generator, allowing the user to control the GAN output through evolutionary exploration of the latent space. However, the traditional GAN latent space presents feature entanglement, which limits the practicability of possible applications of DeepIE. In this paper, we implement DeepIE within a style-based generator from a StyleGAN model trained on the WikiArt dataset and propose StyleIE, a variation of DeepIE that takes advantage of the secondary disentangled latent space in the style-based generator. We performed two AB/BA crossover user tests that compared the performance of DeepIE against StyleIE for art generation. Self-rated evaluations of the performance were collected through a questionnaire. Findings from the tests suggest that StyleIE and DeepIE perform equally in tasks with open-ended goals with relaxed constraints, but StyleIE performs better in close-ended and more constrained tasks.

## 1. Introduction

In 1987, Richard Dawkins introduced a system of simulated selective breeding of artificial organisms called Biomorph [1], becoming a milestone in the birth of interactive evolutionary computation (IEC) and evolutionary art (EArt) as areas of research [2,3]. EArt can be understood as the development and application of evolutionary computation techniques for the generation of computer graphics [2]. Traditionally, evolutionary computation (EC) [4] exerts pressure over the population through a fitness function that evaluates the candidates in order to solve an optimization problem. However, evaluation becomes a challenge in problems where the fitness function results are intractable or subjective, such as creative and artistic tasks. The Biomporh was one of the first examples of evolutionary algorithms with user preference as the selection pressure. Since then, a significant amount of research on IEC was conducted on the generation of graphic art, animation, music, and other forms of EArt [3], as well as more technical applications such as online news aggregation [5], online services [6] and search engines [7]. More novel approaches for evolutionary computation include music recommendation systems based on emotion [8] and community detection [9]. IEC applied to EArt can allow the human user to generate an artistic artifact of complexity and aesthetic value beyond their skill or to explore a novel set of aesthetics. However, IEC still faces some practical problems, such as user fatigue. To sort these issues, the combination of deep generative models and IEC has been proposed in [10]. This novel approach is called deep interactive evolution (DeepIE).

This paper explores the intersection of generative adversarial networks (GANs) and interactive evolutionary computation (IEC) within the context of artistic artifact generation and creative expression. GANs are state-of-the-art generative models that have been applied in artifact generation involving video, audio, 3D models, virtual ambiance and videogames [11,12,13,14], but have found most of their success in the generation of 2D images [15]. GANs generate new artifacts by sampling from a learned latent space; however, this sampling process is mostly done stochastically and offers little control over the final output. IEC is a branch of evolutionary computation (EC) that includes the subjective evaluation of a human user to work in tandem with an evolutionary algorithm to solve an optimization problem. IEC is particularly useful in problems where the solution is subjective, such as art generation [3]. However, IEC still faces some practical problems, principally user fatigue and genotype-to-phenotype mappings that are not conducive to human-guided search. Deep interactive evolution (DeepIE) is a novel approach to artifact generation that combines GANs and IEC in a way that helps to overcome the shortcomings of both [10]. DeepIE uses the generator part of a generative adversarial network (GAN) trained on a specific target domain to map between the genotype and phenotype in an IEC algorithm. On one hand, the search is reduced mostly to desirable artifacts, thus reducing user fatigue. On the other hand, the IEC algorithm allows for directed evolutionary exploration of the GAN latent space. DeepIE was tested in [10] with encouraging results, thereby opening the possibility of employing this tool for creative exploration, particularly in the generation of artistic images. This possibility is supported by the success of Artbreeder [16], a popular collaboratory art generator based on a similar evolutionary exploration of GAN latent space.

We first began exploring the capabilities of DeepIE for artistic production in [17], and while the overall results were positive, we observed that DeepIE presented challenges commonly associated with generative models such as GANs, mainly feature entanglement. Feature entanglement is a phenomenon within generative models that are not capable of mapping parts of the input vector to specific features. Features are then entangled with each other among the input vector and can not be modified individually, making the outputs of such models difficult to control. This problem severely restricts the potential of DeepIE as an artistic tool. As a solution, we theorized that implementing DeepIE alongside a generative architecture resistant to feature entanglement should improve the evolutionary process. Fortunately, such a model already exists in the form of the style-based generator architecture for generative adversarial networks (StyleGAN) [18]. We adapted the DeepIE algorithm to work in the secondary disentangled latent space of the style-based generator in a manner that facilitates user control over the evolutionary process on specific feature styles of the resulting artifacts. We called this new approach deep style-based interactive evolution (StyleIE). As a result of our study, we can now describe the main contributions: this is both the first study that explores the potential of deep interactive evolutionary systems for creative exploration and the first implementation of a DeepIE variant within the style-based generator, taking advantage of the intermediate latent space of the mapping network as a more complex genotype representation to allow crossover operation over disentangled features.

The rest of this paper is structured as follows. After an overview of the theoretical background in Section 2, we illustrate our DeepIE implementation in StyleGAN and our proposed StyleIE algorithm in Section 3. We propose a methodology for the experimental validation by user testing in Section 4 and the results of said testing are shown in Section 5. Section 6 is dedicated to the discussion of results. Finally, Section 7 contains conclusions, limitations, and contributions.

## 2. Background and Related Work

### 2.1. Interactive Evolutionary Computation

IEC can be defined as an evolutionary computation optimization method whose fitness functions are replaced by the subjective evaluation of a human user. IEC can also be understood as a cooperative endeavor between the human user and the EC system that maps the computational parameters with the psychological space in the user’s mind. Interactive versions of most EC techniques such as genetic algorithms, evolutionary strategy, and genetic programming exist. Genetic operators such as crossover and mutation are common in IEC applications, especially in a subtype of IEC know as simulated breeding or user selection [3]. For some optimization problems, it can be difficult or even impossible to design an explicit fitness function. This is the case for systems that produce outputs that must be subjectively evaluated, such as art or music. In those cases, IEC is an effective optimization method, because it has the capacity to embed human preference and subjective perception into the optimization process. In IEC, the human and the EC cooperatively optimize a target based on a mapping relationship between the computational search space of the genotype, the solution search space of the phenotype, and the psychological space of human preference. Individuals are evaluated according to the distance between the imagined target in the psychological spaces and the actual system outputs, with the human acting as a black box evaluator. It should be noted that human preference fluctuates, so in IEC the target is constantly moving, sometimes in response to the output of the EC. Since every output the human user considers to be equally satisfactory or indistinguishable can be considered acceptable as a solution, the global optima is not a point, but an area [3], and not unique [19]. Global optima cannot be narrowly defined in an IEC problem; since computationally different outputs can be equally psychologically satisfactory for the user, it is better to understand global optima in this context as an area rather than a point in the search space. One of the strengths of IEC is that it embeds human preferences and intuition into optimization systems, which is very useful in problems with no tractable fitness function, such as in EArt. Downsides of IEC include the fact that population sizes are limited by the number of individuals the human user could meaningfully evaluate; for this reason, populations in IEC are exponentially smaller than their EC counterparts. Moreover, user fatigue limits the number of search generations [3]. Genotype-to-phenotype mapping, also known as the representation problem in EArt [20], greatly influences user fatigue, user satisfaction, and the resulting artifact [2,10].

### 2.2. Generative Adversarial Networks

GANs were first introduced by Goodfellow et al. [21], rapidly gaining popularity among the machine learning community and being mostly employed in the task of image generation [15]. In the GAN framework, two models are trained simultaneously as adversaries: a generative neural network model *G* that learns the data distribution from a noisy sample, denoted by the letter *Z*, and a discriminative neural network model *D* that learns to distinguish between the original training data and the fake output of *G*. The adversarial training corresponds to a minimax game, where the training goal for *G* is to maximize the probability of *D* making a mistake. The networks compete until a Nash equilibrium is reached; at this point, the fake samples should be indistinguishable from the real data [21]. Both the generator and discriminator can be arbitrary neural networks [22]. The original GAN proposal used fully connected neural networks and Jensen–Shannon divergence to measure the difference between the original and the learned data distribution. Numerous improvements have been built upon the initial architecture, such as employing the Wasserstein distance in place of Jensen–Shannon [23], including gradient penalization to improve training speed [24], convolutional architectures for image generation [24], and batch normalization [25]. Training adversarial models in high resolutions remains challenging: higher resolutions mean that the real and generated images are easier for the discriminator to tell apart. Memory constraints also limit the mini-batch size for large resolution images. Karras et al. [26] introduced progressive training (ProGAN), which grows both networks progressively: starting from a low resolution, new layers are added both on the discriminator and the generator as training progresses. This incremental approach accelerates and stabilizes the process. In lower resolutions, the network learns the large-scale structure of the images, and in higher resolutions, the learning is shifted towards finer details. The generator and discriminator are mirrors of each other and grow simultaneously.

### 2.3. A Style-Based Generator

Style-based generators were introduced by Karras et al. [18] in the StyleGAN architecture. StyleGAN further improves the progressive training of ProGAN by redesigning the generator to adjust the style of each convolutional layer and avoids feature entanglement. ProGAN can generate high-quality images but provides a very limited ability to control the specific features of the generated images. The reason for this is that when the generator learns to represent the training in the latent space, such a representation has to follow the probability density of the training data. Feature entanglement in the latent space happens when the GAN model encodes features into multiple dimensions. This means that specific features can not be modified without modifying other features. A disentangled latent space would consist of linear subspaces, each controlling a specific feature. The major challenge for disentangling latent spaces is that the probability of the latent space should match the density of the training data. The style-based generators introduce a solution for this problem as a mapping subnetwork and an intermediate learned latent space *W*. Ideally, disentangling the latent space means that the dimensions in the latent vectors correspond to no more than one feature. In practice this is still far from being perfectly achieved; however, StyleGAN represents an important step in that direction [27]. The generator consists of two subnetworks: a mapping network and a synthesis network. The mapping network consists of eight fully connected layers and receives its input directly from the Gaussian distribution *Z* with a dimensionality of 512. The synthesis network consists of 18 convolutional layers, two for each resolution (starting from 4×4 up to 1024×1024), and receives a learned constant as an input, instead of sampling from *Z*. The mapping network is a fully connected network that encodes the original latent vector into an intermediate latent space denoted by *W*, which does not have to follow the original probability distribution *Z*. This new vector in the intermediate latent space is called the style vector and is then transformed and incorporated into each block of the generator model after the convolutional layers via an operation called adaptive instance normalization or AdaIN, which encodes the *w* vector from the mapping network into the generated images. An AdaIN module is incorporated into each convolutional layer in the synthesis network, defining the visual expression of the characteristics in that level [27]. AdaIN is a type of instance (or contrast) normalization used for style transfer between images [28]. AdaIN receives the convolutional feature maps of two image inputs (which are tridimensional matrices), a content input *x* and a style input *y*, and performs style transfer from *y* to *x* by aligning the mean and variance of each channel of *x* to match the mean (μ) and variance (σ) of each channel of *y*. AdaIN does not depend on learned parameters; instead, it normalizes the input *x* with σ(y) and μ(y). In other words, AdaIN performs style transfer in the feature space by normalizing the channel-wise mean and variance of specific feature maps given by Equation (1):

(1) $$AdaIN\left(x,y\right)=\sigma \left(y\right)\left(\frac{x-\mu (x)}{\sigma (x)}\right)+\mu \left(y\right)$$

In the context of StyleGAN, the style vector *w* is transformed by a fully-connected layer into scale and bias terms for each channel that corresponds to the σ(y) and μ(y) terms for the AdaIN operator. The AdaIN operator standardizes the feature map output of each convolutional layer into a Gaussian distribution; then scales and bias them using the σ(y) and μ(y) parameters computed from *w*. In StyleGAN, the style *y* is computed from vector *w* instead of an image’s feature map. Thus, the information from *w* is translated to a visual representation, allowing *w* to control styles in the generated images.

In a traditional GAN, the generator receives the latent vector *Z* through the input layer. The style-based generator omits the input layer and starts from a learned constant instead. The latent vector *z* is fed to a non-linear mapping network f:Z→W which produces w∈W. Learned affine transformations then specialize *w* to styles *y* that control the AdaIN on each convolutional layer. Each feature map of *x* is normalized separately and scaled and biased using the components of style *y*. This allows the model to compute spatially invariant styles for each layer for unsupervised style-transfer. This means that different styles of the image are encoded into a specific style latent vector for each layer, starting from the coarse styles of lower resolutions and ending in the fine styles of higher resolutions. Explicit noise is broadcast to all feature maps and added to the output of the convolution, to generate stochastic detail in the resulting images. The input for the generator can be encoded in an 18×512 style matrix, on which the learned styles obtained from AdaIN are encoded into a specific style vector within the matrix and no longer entangled. Each row within the style matrix corresponds to a style vector that controls a specific style in the output image.

**Figure 1:**
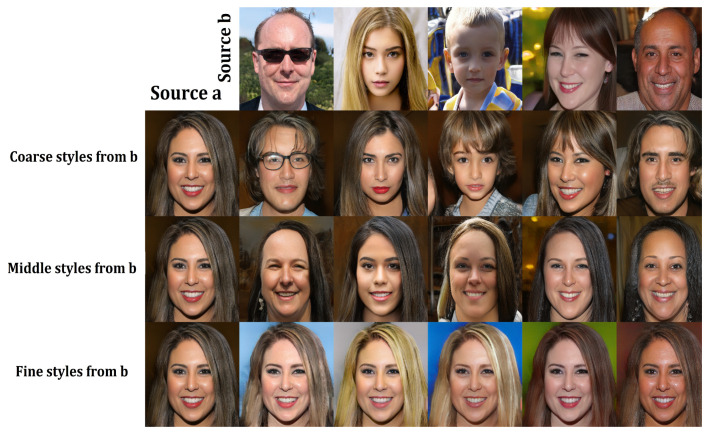
Two sets of images were generated from their respective latent code (sources *a* and *b*); the rest of the images were generated by copying a specified subset of styles from source *b* and taking the rest from source *a*. Copying the styles corresponding to coarse spatial resolutions ($4^{2}$–$8^{2}$) brings high-level aspects of source *b* (such as general face shape and shape) while finer and middle aspects remain from source *a*. Middle resolution styles ($16^{2}$–$32^{2}$) from *b* bring specific details, such as subtle facial details, and fine styles ($64^{2}$–$1024^{2}$) principally bring the color scheme. Adapted from [18].

The synthesis network works on nine resolutions, from 4×4 to 1024×1024. For each resolution, there are 2 convolutional layers, meaning that the network consists of 18 convolutional layers in total. The resulting latent representation in *w* is actually an 18×512 matrix with a row-vector for every convolutional layer that encodes a different feature style of the image. The authors in [18] noted that we can visually identify general characteristics in the encoded styles of *W*, according to the resolution of the convolutional layers involved in their encoding. We can observe that lower resolution layers (the first four row-vectors in the matrix) encode coarse styles. Middle resolution layers (the next four row-vectors) encode “middle styles.” High resolution layers (the last ten row-vectors) encode “finer styles.” This allows for style transfer within *w* latent matrices, as seen in Figure 1.

### 2.4. Deep Interactive Evolution

DeepIE is a novel approach to the artifact generation problem that combines IEC and GANs to avoid some of the disadvantages of traditional IEC [10]. DeepIE follows the structure of a simple interactive genetic algorithm (IGA) [29], where the genotype is the latent vector *z* sampled from a Gaussian distribution fed to the GAN, and the phenotypes are the generated images. DeepIE is a form of simulated breeding that relies on evolutionary operators such as crossover and mutation. The crossover and mutation operators are done at the genotype level, where the components of the latent vector are the alleles. The rationale behind DeepIE is that the trained generator of a GAN can be used as a map between genotype and phenotype within a particular domain. The latent space of the generator becomes the search space of the IGA. The objective of the DeepIE system is to map the search space of the IEC to the psychological search space in the user’s mind. This is the main conceptual appeal of DeepIE compared to other IEC techniques: better artifact representations and a learned search space where every point corresponds to viable artifacts. The generative model produces an initial population of *n* individuals from random latent vectors. The user selects the images of their preference. The latent vectors of the selected images are included in the mating pool. Pairs of images are selected through random choice and (through uniform crossover and mutation) their respective latent vectors are used to create a new population of latent vectors. The new population can be enriched by the introduction of foreigners. The new latent vectors are used as input for the generative model to create a new set of images. The user is then free to select images from the new population and continue the iterative process. The end condition can be either the user’s decision to stop or a maximum number of generations achieved.

However, DeepIE still faces some challenges. The principal problem is that traditional GAN generator usually presents feature entanglement in their latent spaces and vectors. In the context of generating images, this means that single visually recognizable features may be entangled within multiple variables of the latent code. Thus, there is no way to modify the latent code in a way that selectively alters a specific feature. This poses a challenge for conducting genetic operators such as crossover in the latent vectors of the model. For example, a crossover in the genotype of the parents (two latent vectors from *Z*) may not translate into a satisfactory combination in the phenotype of the children. A crossover between latent vectors presenting entanglement may lead to non-intuitive results.

## 3. Solution Model

### 3.1. StyleGAN for Art Generation

For this study, we implemented the DeepIE algorithm to work with the style-based generator of a StyleGAN model trained to generate visual art, particularly of the western academic canon. Two versions were implemented: the original DeepIE, as described in [10], and our modified version for the StyleGAN architecture, which we named StyleIE.

#### 3.1.1. The WikiArt dataset

The WikiArt website has a substantial collection of historical artworks consisting of 80,000 high-resolutionimages that encompass the following styles: abstract art, abstract expressionism, art informal, art nouveau (modern), baroque, color field painting, cubism, early renaissance, expressionism, high renaissance, impressionism, magic realism, mannerism (late renaissance), minimalism, naive art (primitivism), neoclassicism, northern renaissance, pop art, postimpressionism, realism, rococo, romanticism, surrealism, symbolism, and Ukiyo-e [30]. Table 1 shows the distributions of images by artistic style on the WikiArt dataset.

#### 3.1.2. Style-Based Generator on the WikiArt-Dataset

**Figure 2:**
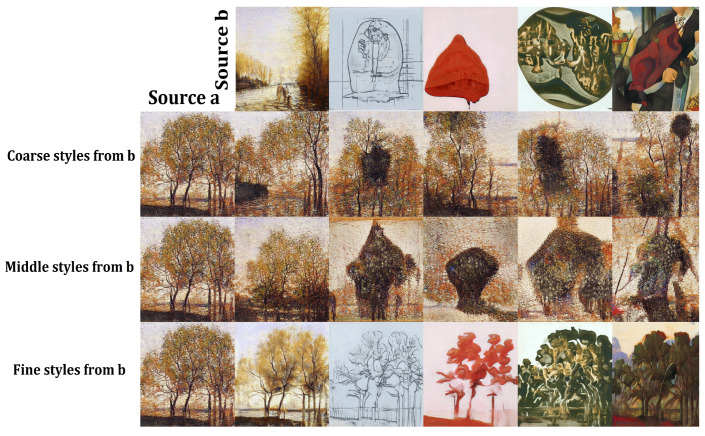
Copying the styles from coarse spatial resolutions ($4^{2}$–$8^{2}$) brings the theme and forms of source *b* while retaining the composition and texture from source *a*. Middle resolution styles ($16^{2}$–$32^{2}$) from *b* to *a* bring the composition from *b* and maintain the forms and texture of *a*. Fine resolutions ($64^{2}$–$1024^{2}$) brings only the color, texture and pictorial style from *b*, while leaving the rest of the image intact.

We trained a StyleGAN model using the WikiArt dataset, maintaining the same parameters and training configurations used in [26]. The resulting images resembled artistic style, and while they may still suffer from some of the distortion endemic to GAN-generated images, those distortions are more problematic in photo-realistic face generation than in the case of neural art. Some authors have even argued that these distortions can be unique components of the aesthetics of neural art [31]. Moreover, the feature separation into three levels of styles observed in [18] still holds in the WikiArt trained model, as seen in Figure 2.

### 3.2. Implementing DeepIE on the Style-Based Generator

#### 3.2.1. Original DeepIE

For this study, we adapted the DeepIE algorithm to work with the style-based generator of a trained StyleGAN model. Our implementation did not differ substantially from the one used in [10] and described in Section 2.4. The population has a constant size of 20 individuals and is randomly initialized. The user can select any number of individuals from the current population to form the mating pool. Pairs of individuals are selected through random choice to serve as parents to the children individuals of the next population; 20 pairs are randomly selected to generate 20 new individuals through crossover and mutation on their *z* latent vectors.

In their work [10], the authors recommend using spherical linear interpolation (slerp) as a crossover operator to maintain the expected distribution of the latent space. Interpolation between latent vectors is a common way of exploring and visualizing the latent space of generative networks [32]. Utilizing it as a crossover operator is the natural extension of this practice for DeepIE. For this reason, we employed spherical linear interpolation (slerp) [33] as the crossover operator for our implementation. Let *a* and *b* be vectors; *t* is the interpolation amount parameter where 0≤t≤1 and Ω=arccos(a·b); the slerp parameter is given by Equation (2):

(2) $$Slerp\left(a,b,t\right)=\left(\frac{\sin\left(1-t)\Omega \right)}{\sin\Omega }\right)a+\left(\frac{\sin\left(t\Omega \right)}{\sin\Omega }\right)b$$

We employed the mutation operator with 50% probability and mediated by a strength parameter set by the user. Mutation is applied as a vector of random noise that is added to the latent vectors. The strength parameter is a coefficient between 0 to 1 that controls the magnitude of the noise vector. We also allowed the user to decide the number of foreign individuals to include in the next population. Foreign individuals take the place of children, so the population size remains constant. In our case, the foreign individuals were generated by randomly sampling from the *Z* latent space. This way the user could obtain novel options for selection in case the population becomes too homogeneous or if it does not include interesting or useful individuals.

**Figure 3:**
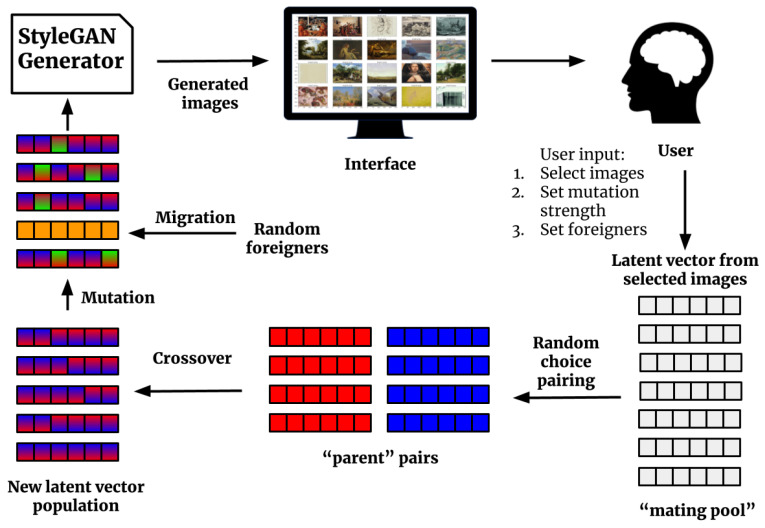
DeepIE Iteration: First, latent vectors enter the StyleGAN generator to produce images. Next, the user selects the images of their preference. Third, the mating pool consists of corresponding vectors of the selected images. Pairs of parents are formed by random choice; enough pairs are formed to generate a complete new population. Fourth, the new population of latent vectors is created through the crossover between the parent pairs. Then the population is mutated and foreign individuals are introduced through migration. Last, the new population of latent vectors enters the generator to produce new images.

In our DeepIE implementation, we treat the style-based generator as any other black box GAN generator. StyleGAN accepts latent input vectors *z* of 512 m length. If we assume the rest of the model as a black box, the output of the model is a 1024×1024 image. The vector input then takes the role of the genotype and the resulting image becomes the phenotype. Using this approach, the DeepIE allows us to treat each variable in vector *z* as an allele and apply genetic operators on them. First, the generative model produces an initial population *Z* of *n* individuals from random latent vectors sampled from the normal distribution $N(\mu ,\sigma^{2})$. Then, a population of images is generated by G(Z). The user selects the images *S* of their preference, which adds to the mating pool. The next step is to create a new generation *Z* of latent vector individuals *z*. For every *z* in *Z* we randomly choose two parents from the mating pool and pair them to generate a new child. This child is generated by slerp crossover between the parents and then receives mutation with a 50% probability, mediated by the strength parameter set by the user. The user selects a number of foreigners *F* sampled from $N(\mu ,\sigma^{2})$ to be introduced in the new generation, taking the place of a child, so the size *n* remains constant. The new population of latent vectors *Z* is used to generate a new set of images G(Z). The user is free to continue the iterative evolutionary process until he decides to stop or a maximum number of generations is reached. Algorithm 1 and Figure 3 outline our implemented DeepIE iterative process.

#### 3.2.2. Style-Based DeepIE

**Figure 4:**
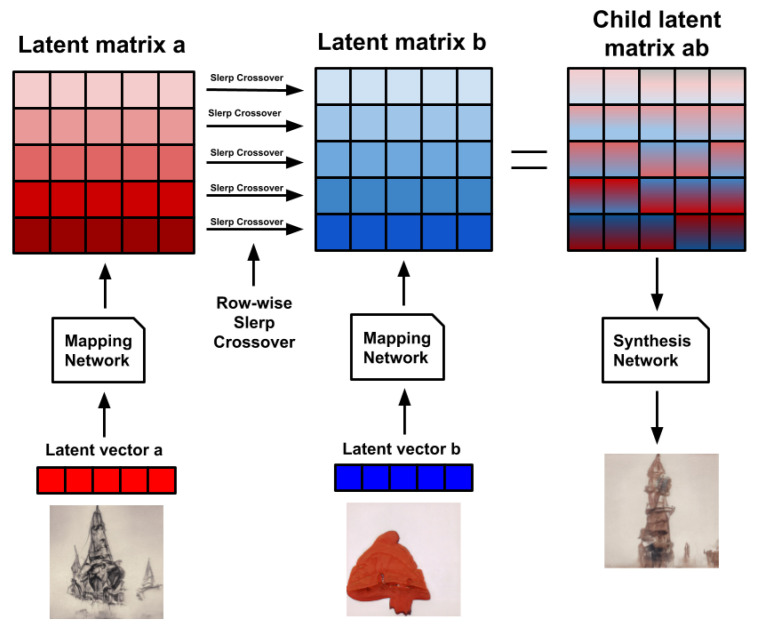
StyleIE crossover diagram. In StyleIE, individuals are two-dimensional latent matrices of size *l* × *m*. Every row $l_{i}$ of length *m* encodes a particular style of the resulting image. When applying crossover between two *w* individuals, crossover should only be applied between corresponding $l_{i}$ rows. Let *a* and *b* be our *l* × *m* parent individuals. This means that every row $l_{i}$ of child ab will be generated by the crossover of row $l_{i}$ in *a* with row $l_{i}$ in *b*, for every row $l_{i}$ to $l_{l}$.

The literature reports that giving users the ability to focus the evolution on a particular phenotypic feature of the individuals can be beneficial for the performance of IEC algorithms [34,35]. For this reason, we believe that DeepIE could benefit from the particular architecture of the style-based generator and offer the user more direct control over specific image features. In our DeepIE implementation, the population *Z* of individuals in a generation is an n×m matrix where *n* is the number of individuals and *m* is the length of the latent vectors. In our proposed modification, the population of individuals *W* is an n×l×m matrix where *n* is the number of individuals, *l* is the number of style vectors and *m* is the length of those vectors. This opens the way to, instead of naively applying the crossover operator on the latent vector *z*, applying the crossover at the intermediate matrix *w*. We applied the operator row-wise among the two style matrices of two parents (see Figure 4). In StyleIE, individuals are two-dimensional latent matrices of size l×m. Every row $l_{i}$ of length *m* encodes a particular style of the resulting image. This means that when applying crossover between two *w* individuals, crossover should only be applied between corresponding $l_{i}$ rows. Let $w_{a}$ and $w_{b}$ be our l×m parent individuals. Every row $l_{i}$ of child $w_{ab}$ will be generated by the crossover of row $l_{i}$ in $w_{a}$ with row $l_{i}$ in $w_{b}$, from $l_{i}$ to $l_{l}$. This way we ensure that the crossover operator is only applied between vectors that encode the same feature style, so features will not be merged across different styles in the resulting images. In other words, while in the original DeepIE the genotype is a vector of 512 dimensions, in our version, the genotype is a matrix of 18×512 (however, it should be noted that the crossover operations between two genotypes should only be done between rows of the same level).
**Algorithm 1:** DeepIE. Defaults: m←512,n←20,p←0.5, G← trained StyleGAN generator.
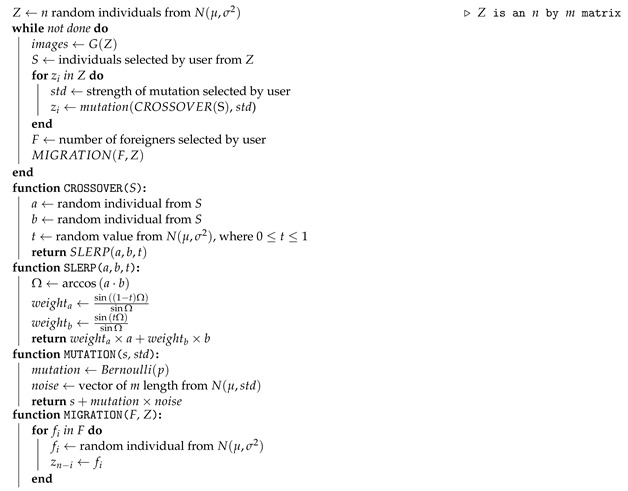


**Figure 5:**
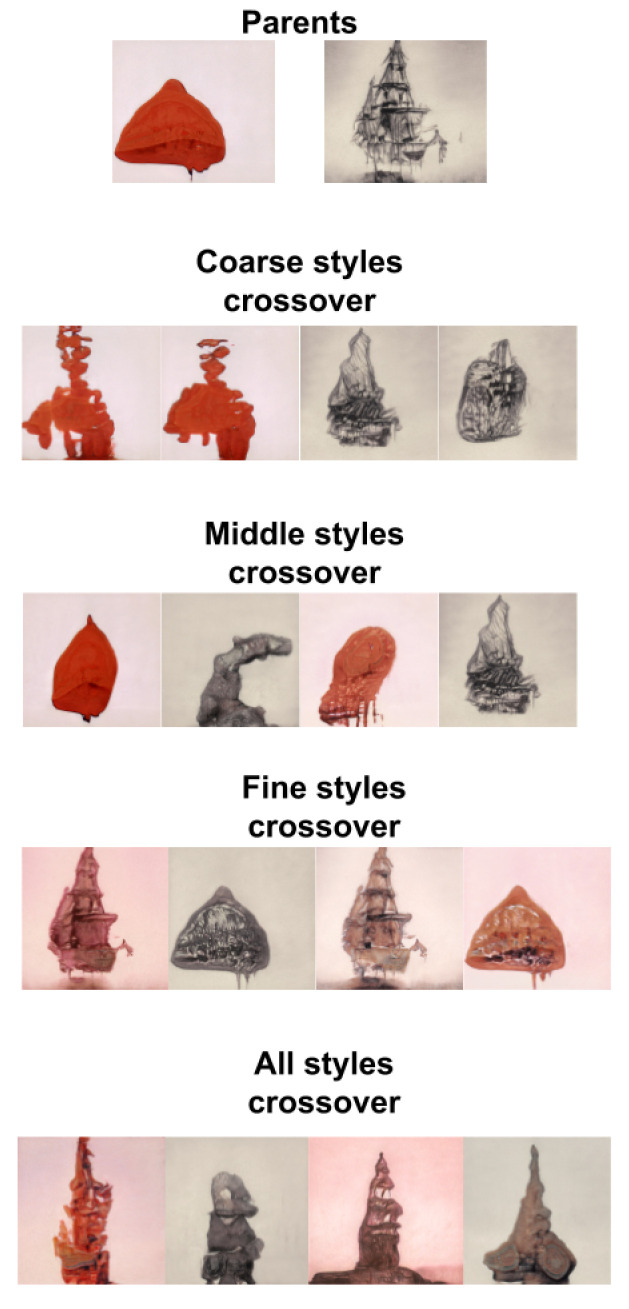
Crossover operations over coarse, middle, fine and all styles.

Performing the crossover operators row-wise over the style vector of the *w* matrices allows the user to have directed control over what feature to evolve. By restricting the crossover operator to a particular set of style vectors, we can either manipulate coarse, middle, and fine styles separately or evolve the image as a whole (see Figure 5). In our implementation, for every new child created in a new generation, the crossover had 25% of probability to be restricted to coarse style, 25% to middle styles, 25% to fine styles and the final 25% of probability to be applied to all styles. We called this new algorithm style-based DeepIE or StyleIE. Algorithm 2 outlines the StyleIE iterative process.


**Algorithm 2:** StyleIE. Defaults: m←512, n←20, l←18, p←0.5, Mn← mapping network of StyleGAN.

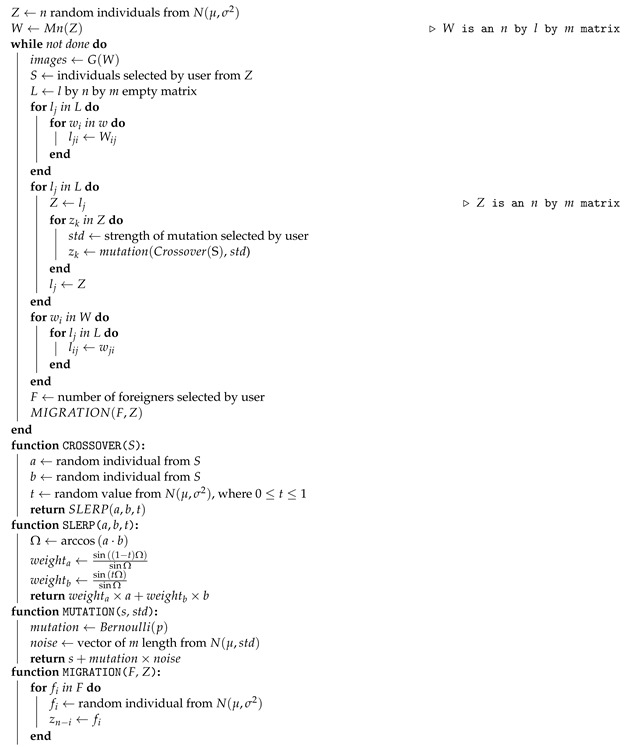




## 4. Methodology

### 4.1. Experimental Design

**Figure 6:**
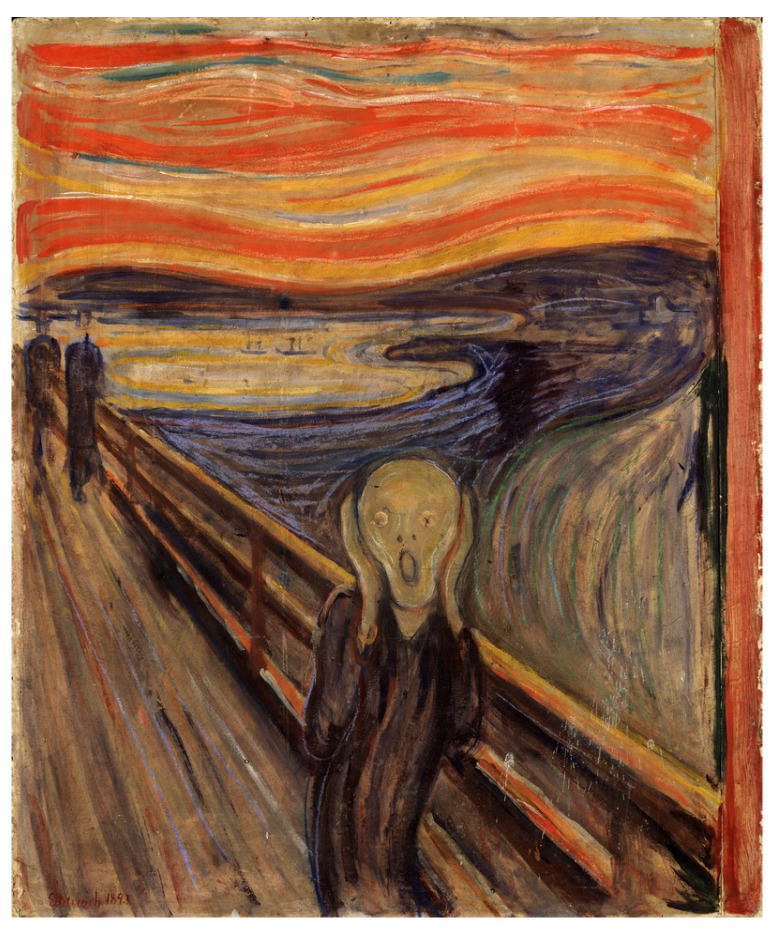
“The Scream” by Edvard Munch (1893).

The experiments in [10] show that it is feasible to use DeepIE to guide the evolution of a population of GAN-generated images in order to approximate a specific target. Our interest was to study the potential of both DeepIE and StyleIE for art generation and creative exploration. We also wanted to evaluate how they compared to each other. In order to do this, we designed two user test experiments to evaluate and compare the performances of the algorithms:

1. In the first experiment, volunteers were tasked to generate an artistic image belonging to a determined genre and artistic style—in this case, an expressionist portrait. The rationale behind this choice was to give the users a set of constraints to direct their search, while still being open enough to allow them room for creative and artistic expression. The expressionist portrait goal was chosen because expressionism is a distinctive artistic style that is neither under nor overrepresented in the training dataset (see Table 1). Thus, finding an instance of expressionist art in the search space should be neither too easy nor too hard. On the other hand, portrait is one of the most straightforward genres in painting, so we reduced some of the ambiguity inherent to artistic tasks. Moreover, portrait is the most common paint genre in the WikiArt dataset [36].
2. In the second experiment, volunteers were tasked to generate an artistic image resembling a specific example, thereby introducing stronger constraints to the task. Instead of asking them to generate an open-ended expressionist portrait, we asked them to approximate a specific one: “The Scream” by Edvard Munch (see Figure 6). It should be emphasized that the instruction was not to replicate the target image but to approximate it instead.

For both experiments, we asked the volunteers to perform the evolutionary process until they believed they had created the most satisfactory image. Once they had finished, they were asked to choose, from the pool of all selected images in every generation, the image they considered to be the most successful for the assigned task. To keep the duration of the activity within a reasonable time frame, we limited the experiments to a maximum of 20 generations. Due to the fact that both experiments required familiarity with artistic styles and genres, we decided that volunteers with artistic backgrounds would be best suited for successfully completing the tasks. For this reason, we recruited senior undergraduate students from the School of Architecture, Art and, Design at Tecnológico de Monterrey. The volunteers had familiarity with artistic styles and genres; however, they did not have any previous experience with evolutionary interactive tools. For this reason, before the experiment, we provided them with a video tutorial on how to use our system.

#### 4.1.1. The AB/BA Test Design

We performed a user test experiment with an AB/BA crossover design. AB/BA testing is a subtype of AB testing borrowed from medical literature [37]. AB tests are a type of simple controlled experiment. In this kind of experiment, two versions of a system are compared. These tests are useful for researching user experience and satisfaction. Version A is usually the current version (DeepIE), while version B is modified in some respect (StyleIE) [38]. In typical AB testing, users only see one of the two versions. In the AB/BA test, a subgroup of users first test version A and then switch to version B, while another subgroup starts with version B and then switches to version A. With AB/BA we can test how the two versions compare to each other while observing if there are any differences in performance that can be accountable for learning effect or user fatigue. It also has the advantage of being able to directly ask the user what version of the algorithm they prefer.

**Figure 7:**
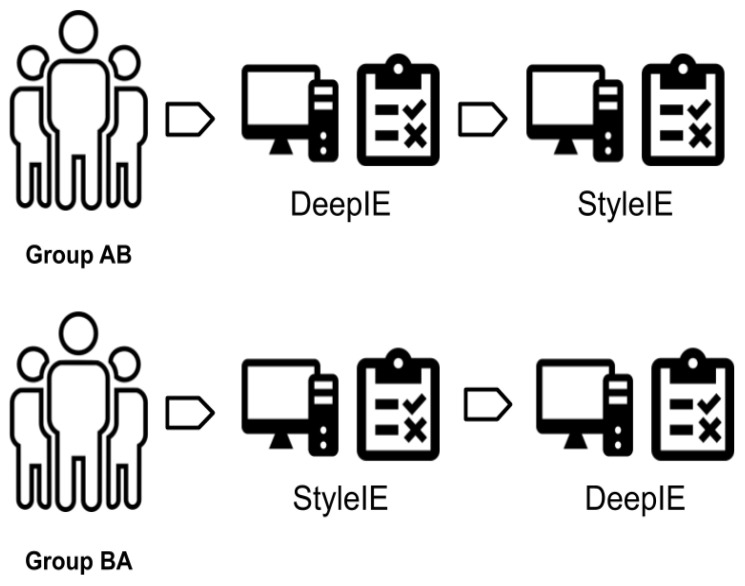
AB/BA test: The users are divided into two groups: The AB group first completes experiment A (DeepIE) and then completes experiment B (StyleIE). The BA group starts with B and continues with A.

We recruited 60 volunteers and divided them into two groups. Group 1 consisted of 20 volunteers that were assigned to experiment 1. Group 2 consisted of 40 volunteers assigned to experiment 2. These groups contained enough individuals to yield valid results since Nielsen and Landauer [39] concluded that the optimal number of test users in user testing is 20. Following the AB/BA testing design, we divided both groups into two subgroups of half the size of the original groups. These two subgroups were denominated AB and BA. AB first performed the assigned task using DeepIE, then performed it a second time using StyleIE. BA first used StyleIE, then switched to DeepIE (See Figure 7). Thus, each user in both experiments completed the assigned task two times, using a different deep interactive evolutionary system each time. After finishing the tasks, the users were asked to select the image they considered to be the most successful and to fill a questionnaire about their experience.

#### 4.1.2. The Questionnaire

IEC is a technique where a human user and an EC cooperatively optimize a target system. For this to be possible, there must exist a mapping relation between the computational search space of the EC and the psychological solution space in the user’s mind. Users evaluate the individuals according to the perceived distance between the target in their psychological space and the actual output of the system. The EC searches for an optimal solution guided by that psychological distance [3]. From this, it follows that the performance and success of an IEC system are highly dependent on the robustness of the mapping between both the computational and psychological search spaces. However, measuring and evaluating this relation can be challenging, since both involve the subjective perceptions of the users [29]. For this reason, it is often necessary to employ subjective tests and evaluations when measuring the success of IEC. Subjective self-evaluation through questionnaires is standard practice among IEC research, and questionnaires similar to our own have been used in IEC literature [10,29,34,40,41]. To assess user performance and perception of our systems, we asked them to answer the following questionnaire:

- Answer the following question with respect to version A.
  - Rate on a scale from 1 to 5 your success in the task.
  - Rate on a scale from 1 to 5 how you perceive the usefulness of the program for artistic and creative expression.
- Answer the following question with respect to version B.
  - Rate on a scale from 1 to 5 your success in the task.
  - Rate on a scale from 1 to 5 how you perceive the usefulness of the program or artistic and creative expression.
- Answer the following question with respect to both versions.
  - Which version do you consider better? Why?
  - Did you employ any kind of strategy for achieving the goal? Explain.
  - Do you have any observations about the creative process of the system?

Our questionnaire consisted of seven questions divided into three sections. The first section is related to version A of the system employed and consisted of two rating questions. The second section is similar to the first one, but belonging to version B instead. The third section asked the user to take into account their comparative experiences with both versions and three open questions were included. For this section, we were mainly interested in assessing which system was preferred by the users. Part of our questionnaire was similar to the one used in [10] since we were asking them to self-rate their success on the same discrete scale. However, we also asked them to rate how they perceive the usefulness of the systems for creative expression.

#### 4.1.3. The Interface

The experiments were conducted remotely using Google Colab Notebooks. A simple interface was implemented to display the current population and allow the user to select the preferred individuals, and set the mutation strength and the number of foreigners for each generation. Our Colab Notebooks and implementation code can be consulted in the Supplementary Material.

## 5. Analysis of Results

### 5.1. Experiment 1

**Figure 8:**
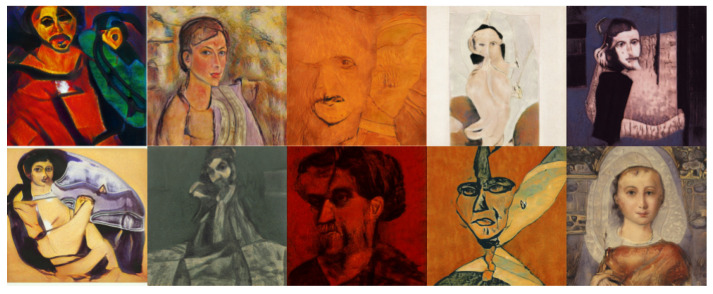
Best images from the AB group using DeepIE.

**Figure 9:**
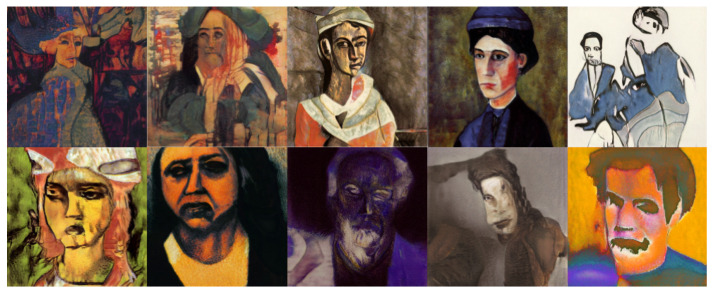
Best images from the AB group using StyleIE.

**Figure 10:**
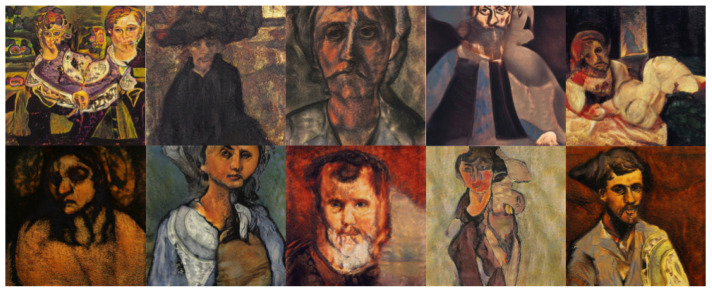
Best images from the BA group using StyleIE.

**Figure 11:**
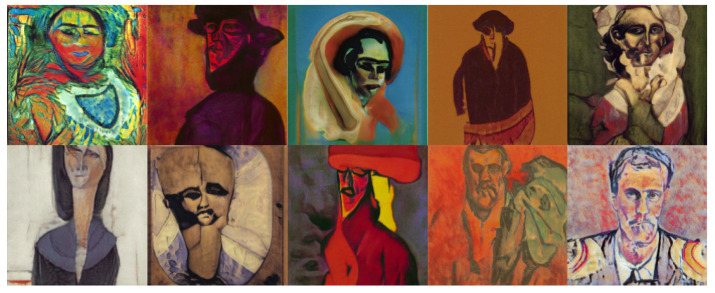
Best images from the AB group using DeepIE.

The objective of this experiment was to evaluate and compare the performances and results of DeepIE and StyleIE in an open-ended problem with relaxed constraints. For this experiment, we recruited 20 volunteers with artistic academic backgrounds. They were divided into two subgroups, AB and BA, of 10 subjects each. The users in each subgroup were asked to generate an expressionist portrait two times. AB generated the first portrait with DeepIE and the second one with StyleIE. BA used the systems in reverse order. The users persisted in the task until they believed they had found a satisfactory image that fulfilled the requirements but had an upper limit of 20 generations to achieve it. After generating the two images, they answered the questionnaire and chose which of all the selected images from every generation was the most satisfactory. The resulting images can be seen in Figure 8, Figure 9, Figure 10 and Figure 11. For comparing the means of obtained survey results between each group, we employed t-test and analysis of variance (ANOVA) using the standard α level of 0.05. Our raw data, as well as t-test and ANOVA calculations for this experiment, can be consulted in the Supplementary Material.

#### 5.1.1. Quantitative Survey Analysis

**Figure 12:**
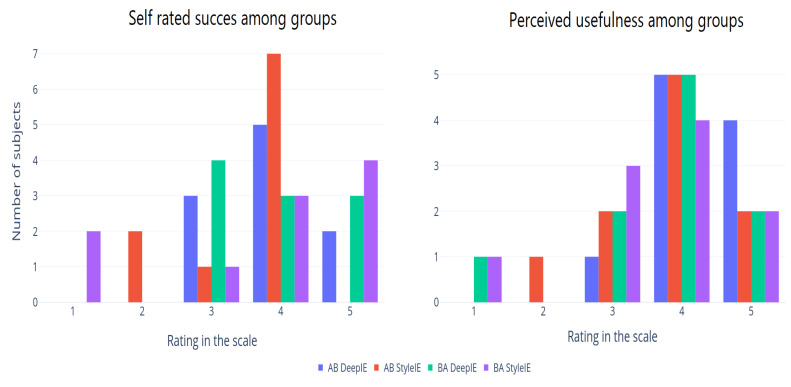
Survey results from experiment 1.

We divided our volunteers into two groups following the AB/BA crossover design. Each group consisted of 10 subjects. The AB group was asked to generate expressionist portraits using DeepIE. Then they were asked to perform the task again using StyleIE instead. The BA group performed the same tasks but switching the order of the systems. After completion, both groups were asked to answer a survey. In the survey, they self-rated (on a scale from 1 to 5) their success in achieving their goal with the system and how useful they considered the system to be (1 to 5). Figure 12 shows the self-rated success among groups and the perceived usefulness in experiment 1. The means of these values are summarized in Table 2 across user groups and systems. ANOVA test showed no significant difference between these groups.

**Figure 13:**
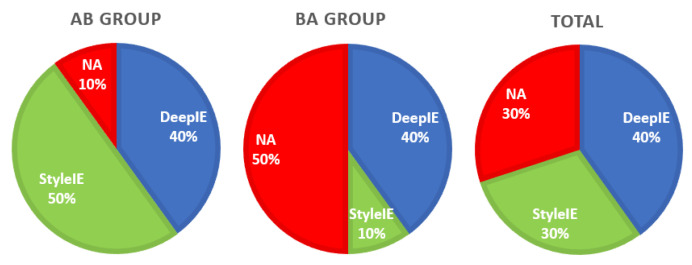
Preferences by group.

In the survey we, also asked the participants to express the preference or ambivalence for any of the systems. Results do not seem to strongly favor any specific system, as shown in Table 3 and Figure 13.

#### 5.1.2. Generations Ratio

**Figure 14:**
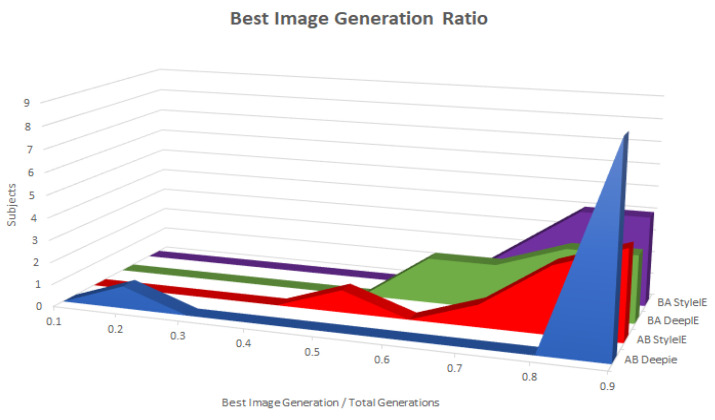
Best image generation ratio among groups.

To gain insight into how far into the evolutionary process the best image occurred, we also measured the ratio between the generation the best image was found in and the total number of generations. Figure 14 shows that the ratios are clustered close to 1, meaning that the image continued to improve throughout the experiment. This is in accordance with the findings of [10], who obtained similar results when evolving human faces with DeepIE. ANOVA test did not find statistically significant differences among the ratios on both groups and methods.

Table 4 shows the mean values of total generations, generation of the best image, and the ratio between them across both groups and methods.

When comparing the performance of the same individual when using DeepIE and when using StyleIE, we employed a paired t-test. We found no significant difference between the total number of generations between the two systems. The same was the case with the generation of the best image found. However, the BA group arrived early to the best image and had an overall lower number of generations with both systems. While the t-test indicates a slight statistical difference among both groups, the variances in those metrics were significantly higher in group AB, meaning that this could be just the effect of an outlier (See Table 5).

We found no statistical evidence that the order of the experiments altered the results of the survey or the duration of the process, suggesting that neither user fatigue nor learning effect were major issues. We then proceeded to aggregate the results of DeepIE and SytleIE in both groups (See Table 6 and Table 7). We found no statistically significant difference between the performance of DeepIE and StyleIE for experiment 1.

#### 5.1.3. Qualitative Survey Analysis

In the survey, we asked the participants to explain the reason for their preference for one system over the other or the lack thereof. Two participants reported that they chose the second system they tried because of their accumulated practice at that point, suggesting that learning effect had some impact on their decision. More “integration of the images,” precision, and practicality were mentioned as the reasons for preferring StyleIE. A more smooth “flow of the images” and more adaptability were listed as well as a reason for preferring DeepIE.

In [10], the authors identified three strategies that surged spontaneously from the user while using DeepIE:

- Collect distinct traits: Identifying distinct traits of the target and selecting images that present these traits. The process is continued until the desired traits are merged.
- Select best likeness: Identifying images that overall resemble more closely the target.
- Hierarchical trait selection: Similar to the first strategy, but focusing on one single trait at a time.

We were curious if similar strategies would also naturally arise in our experiment, so we asked in the survey if the participant had used some kind of strategy. Five users reported strategies that resembled collect distinct traits and six users select best likeness. Interestingly, four out of five users that used collect distinct traits preferred StyleIE, while four out of six users that reported select best likeness preferred DeepIE. This could indicate that strategies based on traits can be more suitable for StylyIE, while strategies based on general likeness work better on DeepIE. The rest of the users could not identify any particular strategy.

### 5.2. Experiment 2

**Figure 15:**
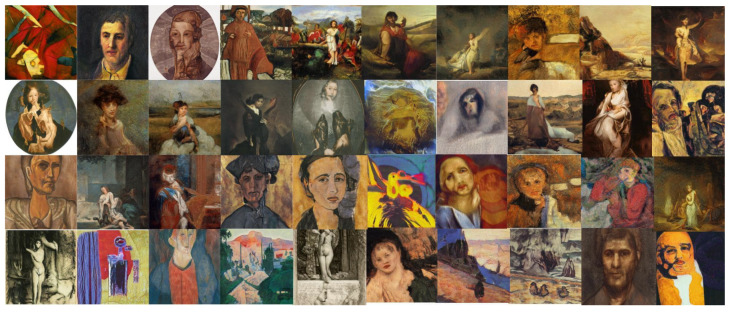
Image selected as the most successful by the users in experiment 2 using DeepIE.

**Figure 16:**
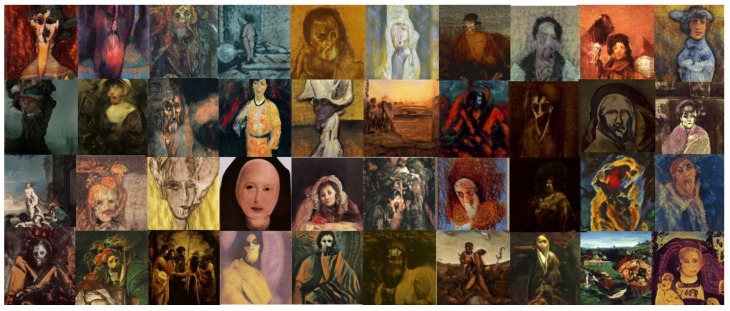
Image selected as the most successful by the users in experiment 2 using StyleIE.

The objective of this experiment was to evaluate and compare the performances and results of DeepIE and StyleIE in a closed-ended problem with strict constraints. For this experiment, we recruited 40 volunteers with an artistic academic background. They were divided into two subgroups AB and BA, of 20 subjects each. The users in each subgroup were asked to approximate the famous expressionist painting “The Scream” by Edvard Munch. As in experiment 1, the AB group first completed the task with DeepIE, and then did it again with StyleIE. The BA group switched the order of the systems. The users persisted in the task until they believed they had found a satisfactory image that fulfilled the requirements but had an upper limit of 20 generations to achieve it. After generating the two images, they answered the questionnaire and chose which of all the selected images from all generations was the most satisfactory. The resulting images can be seen in Figure 15 and Figure 16. Our raw data, as well as t-test and ANOVA calculations for this experiment, can be consulted in the Supplementary Material.

#### 5.2.1. Quantitative Survey Results

**Figure 17:**
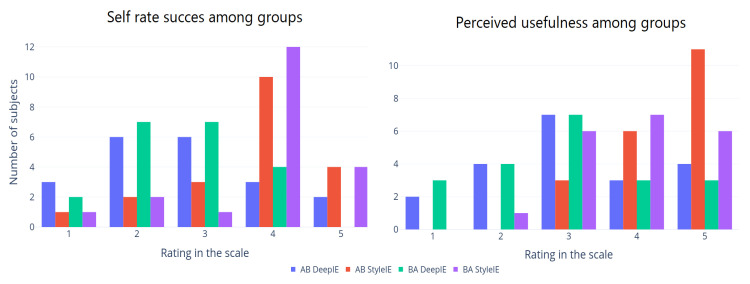
Survey results from experiment 2.

After completion of experiment 2, both groups AB and BA were asked to answer a survey. In the survey, they self-rated (on a scale from 1 to 5) their success in achieving their goal with the system and how useful they considered the system to be (1 to 5). Figure 17 shows the self-rated success among groups and the perceived usefulness in experiment 2.

Table 8 shows the mean scores of self-rate success and perceived usefulness for both groups and systems. To measure whether the order of use had any impact on the reported results, we first performed ANOVA testing among groups and systems in the scores of self-rated success and perceived usefulness. ANOVA testing showed a statistically significant difference between the means. Then we proceeded to compare the results of AB DeepIE with BA DeepIE and AB StyleIE with BA StyleIE. In order to do this, we used an independent t-test, since the compared results were from different individuals. We found no statistically significant differences in any of the means, except in the perceived usefulness of StyleIE with both groups. The implications of these results are discussed in Section 6.

To measure how both systems compared to each other in the survey results, we compared the rating scores among DeepIE and StyleIE in the same group. StyleIE presented higher mean scores for both self-rated success and perceived usefulness in both AB and BA (See Table 9 and Table 10). Since we compared results within the same users, we employed paired t-test. Results from t-tests showed that there is a statistically significant difference in the rating of both systems among the two groups.

We proceeded to aggregate the results of both groups and compare the means. The mean ratings of questions for StyleIE were higher than those of DeepIE (See Table 11). Paired t-tests showed that the differences between those means were statistically significant.

**Figure 18:**
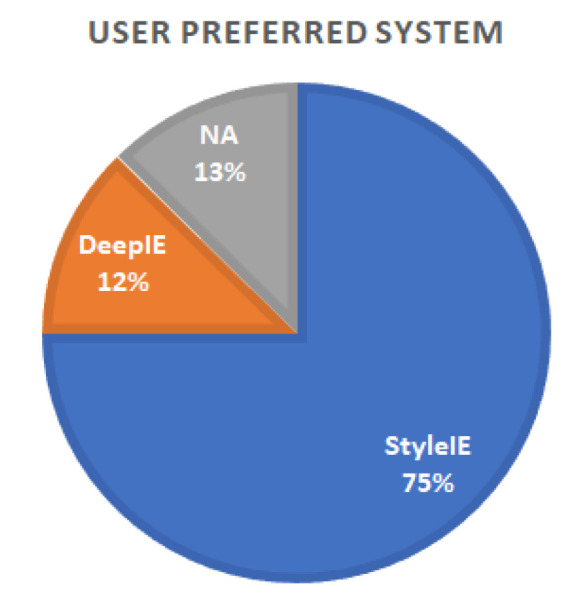
Preferences by system.

As in experiment 1, we also asked the participants to express the preference or ambivalence for any of the systems. Five participants reported preferring DeepIE, 30 participants preferred StyleIE and five had no preference; 75% of test users expressed preference of StyleIE over DeepIE or no preference (see Figure 18).

#### 5.2.2. Generations Ratio

**Figure 19:**
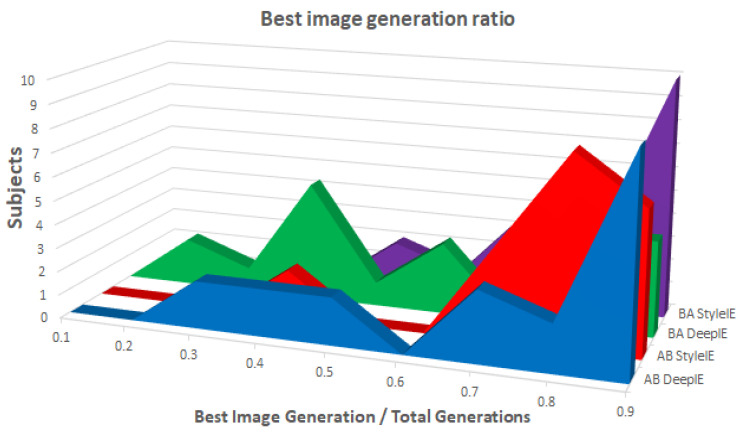
Best image generation ratio among groups.

As in experiment 1, we kept track of the ratio between the generation of the best imaged that was generated and the total number of generations. Figure 19 shows the distribution of ratios in both groups with the two systems. ANOVA test found a statistically significant difference between the means of the groups and systems. Table 12 shows the mean values of total generations, generation of best image and the ratio between them across both groups and systems.

We compared the total number of generations, the generation of the best image found, and the ratio between them with both DeepIE and StyleIE in the AB group using paired t-tests. We found no statistically significant differences between the means of total number of generations, the generation of best image, and ratio of DeepIE to StyleIE in the AB group. In the BA groups, there was no statistically significant difference in the total number of generations of DeepIE and StyleIE, but we found a statistically significant difference in the means of the best generations found and the ratio. StyleIE found the best image later than DeepIE, but its ratio was closer to 1. The authors in [10] interpreted a ratio close to 1 as a sign that images kept improving the longer the evolutionary process was continued. When we aggregated the results of both groups we found no statistical difference in the total number of generations and the generation of the best image found in DeepIE and StyleIE. However, there is a statistically significant difference in the ratios of both systems. The ratios in StyleIE were closer to 1 than those in DeepIE.

#### 5.2.3. Qualitative Survey Analysis

In the qualitative part of the survey, users expressed their opinion about the systems. We asked them to explain their reasons for a preference of one system over the other. We showcase some of the feedback reported:

- A participant commented that they preferred StyleIE because it reflected better their intention when trying to combine the image.
- Another expressed that StyleIE led to more interesting variations and could be useful to explore novel ideas.
- Another expressed that StyleIE was more predictable.
- A user noted that while he preferred DeepIE, he often found that an element he wanted to preserve got lost.

We also asked them about the strategies that they employed when conducting the evolutionary process. Most users reported using a strategy resembling collect distinct traits or hierarchical trait selection, regardless of their preferred system.

## 6. Discussion

We found that users performed equally using DeepIE and StyleIE in an open-ended task with relaxed goal constraints, but StyleIE outperforms DeepIE in a closed-ended task with strong goal constraints. In the first experiment, the totality of users showed no strong preference for either system, suggesting that both were equally effective in aiding them towards the desired goal. In the second experiment, 75% preferred StyleIE over DeepIE, suggesting that StyleIE is more useful the more specific the target. Our self-rated success mean scores in both experiments surpass those reported in [10] for generating a specific celebrity face (mean score of 2.2 for self-rated success) and are similar to those obtained for generating a shoe design (mean score of 3.8 for self-rated success). This could be because modeling a human face using DeepIE could be very challenging since they are operating in an entangled latent space. However, since the tasks evaluated are different and [10] did not provide the raw data of their experiments, we can not validate the similarity of our results with theirs using ANOVA testing. Thus, we should take any similarity between them cautiously.

The scores for the perceived usefulness of the system were generally good in experiment 1 for both systems. In experiment 2, the scores favored StyleIE. We found no correlation between self-rated success and perceived usefulness, meaning that the users judged the usefulness of the system independently of their perceived performance with it. In the qualitative part of the survey, users indicated interest and excitement for the evolutionary system, which in conjunction with the reported usefulness, suggests that deep interactive evolutionary systems have a potential market and applications in the creative industries.

According to the best image generation ratios in experiment 1, the best image was usually found in the last generations, which is a positive indicator that the subjective quality of images kept improving for the users. This indicates that the systems are conductive for user-guided search and that the systems have the potential to keep improving the images in subsequent generations. In experiment 2, ratios were closer to 1 when using StyleIE than when using DeepIE. This suggests that StyleIE is more conducice to the search when the goal is more strongly constrained.

Survey results in experiment 1 indicate that preference for one or another system may be correlated with the chosen strategy of each user. Most users that preferred StyleIE used strategies based on collecting specific traits in the desired image, reporting a more precise robust combination of the traits. On the other hand, users that preferred DeepIE reported a more steady flow of exploration and employed strategies based on general likeness. This can be interpreted as that DeepIE could be more suitable for best likeness strategies that evolve the generality of the image and StyleIE could favor trait selection that offers the possibility of fine-tuned evolution. However, experiment 2 seems to contradict this idea, since most users preferred trait-based strategies regardless of their preferred system. An explanation for this could be that in experiment 1, the relaxed constraints gave the users more freedom to explore different strategies, while the stronger constraint of experiment 2 prompts the users to employ trait-based strategies. This is an intriguing matter indeed and more research on the optimal strategy for deep interactive evolutionary systems should be worthwhile.

## 7. Conclusions

IEC can be used to embed the user’s preference as guidance for a search algorithm and deep generative models can learn distributions of large collections of human knowledge and artifacts to later synthesize plausible instances from them. DeepIE was proposed as a new paradigm for combining IEC with deep generative models; however, its suitability for creative and artistic exploration remained to be evaluated under research conditions.

This study implemented DeepIE with the style-based generator of a StyleGAN model trained to produce works of visual art. We discovered that the style-based generator allowed us to implement DeepIE in a novel way that takes advantage of the disentangled intermediate space in the mapping network, allowing us to perform genetic operators among specific feature style vectors. We named this modification StyleIE.

Finally, we tested the performances of DeepIE and StyleIE in two experiments. The first experiment consisted of a task with relaxed goal constraints. The second experiment had a more strongly constrained goal. DeepIE and StyleIE performed equally under relaxed constraints. StyleIE outperformed DeepIE under stronger constraints. Due to its style-based architecture, StyleIE provides more control over specific features of the generated images. Under the relaxed constraints, the area of the global optima is wider, meaning that more individuals satisfy the constraint requirements and there is no need for fine control over specific features. Therefore, StylIE offers no advantage to DeepIE under those conditions. However, if we constrain the problem, the area of the global optima narrows, meaning that fewer individuals are acceptable solutions. In this case, control over specific features offers a significant advantage to the users.

Research on deep interactive evolutionary systems is still in its infancy. However, we have obtained preliminary evidence that deep interactive evolutionary systems have potential applications in creative and artistic tasks, and that our proposed method, StyleIE, offers a significant advantage over DeepIE under certain goal conditions. This could lead to developing interesting applications in diverse fields related to the creative industries such as design, art, and multimedia projects.

### 7.1. Contributions

- To our knowledge, this is the first study that explores the potential of deep interactive evolutionary systems for creative exploration using students from a school of architecture, art, and design.
- This is also the first study to implement DeepIE within the style-based generator, taking advantage of the intermediate space of the mapping network to produce a more complex genotype and offering the possibility of applying crossover over disentangled features.

### 7.2. Limitations

The user interface constitutes a huge limitation, as was also noted in [10]. A more amicable design may improve the performance and commodity of the users. More user-friendly features, such as the ability to save promising items for later use and a ranking system that prioritizes certain items in the “mating pool” could provide a better experience for the users. On the other hand, our questionnaire was rather simple in its design and scope. We did not employ cross-check questions or redundant questions to validate the consistency and coherency of the answers.

We centered our experiments around a particular artistic style, expressionism. This was done for practical and time limitations. While we did not test the performance of the systems when optimizing for other styles, in principle, there is no reason to believe that the results should not generalize. As long as the target style in question is present in the training data, it should be possible to guide the evolutionary process towards it.

Another limitation is that we did not evaluate the usability of the tool. We leave open the possibility of addressing this issue in future work.

## Figures and Tables

**Table 1:** Images by artistic style on the WikiArt dataset.

Art Style	Number of Images	Percentage
Color Field Painting	1258	1.53
Surrealism	4799	5.84
Neoclassicism	2700	3.28
Realism	10,090	12.28
Baroque	3992	4.86
Romanticism	6569	7.99
Rococo	1928	2.34
Art Informel	969	1.17
Magic Realism	1011	1.23
Symbolism	4063	4.94
Naive Art (Primitivism)	2026	2.46
Abstract Expressionism	2091	2.54
Pop Art	1129	1.37
Expressionism	5844	7.11
High Renaissance	1275	1.55
Impressionism	121,641	14.81
Minimalism	1267	1.54
Cubism	1686	2.05
Abstract Art	1006	1.22
Post-Impressionism	5998	7.30
Mannerism (Late Renaissance)	1192	1.45
Northern Renaissance	2405	2.92
Art Nouveau	4163	5.06
Ukiyo-e	1178	1.43
Early Renaissance	1330	1.61
Total	82,133	100

**Table 2:** Group survey means.

	Self-Rate Success	Perceived Usefulness
AB DeepIE	3.9	4.3
AB StyleIE	3.5	3.8
BA DeepIE	3.9	3.7
BA StyleIE	3.7	3.6

**Table 3:** Preferences of participants.

	AB Group	BA Group	Total
DeepIE	4	4	8
StyleIE	5	1	6
NA	1	5	6

**Table 4:** Generation means.

	Total Generations	Generation of Best Image	Ratio
AB DeepIE	10.7	8.3	0.794
AB StyleIE	10.4	7.9	0.783
BA DeepIE	8	6	0.750
BA StyleIE	7.4	5.8	0.783

**Table 5:** Generation variances.

	Total Generations	Generation of Best Image
AB DeepIE	19.78	20.67
AB StyleIE	17.6	6.766
BA DeepIE	4.66	3.33
BA StyleIE	1.15	1.28

**Table 6:** Aggregated survey means.

	Self-Rate Success	Perceived Usefulness
DeepIE	3.9	4
StyleIE	3.6	3.7

**Table 7:** Aggregated generation means.

	Total Generations	Generation of Best Image	Ratio
DeepIE	9.35	7.15	0.772
StyleIE	8.9	6.85	0.783

**Table 8:** Group survey means.

	Self-Rate Success	Perceived Usefulness
AB DeepIE	2.75	3.15
AB StyleIE	3.7	4.4
BA DeepIE	2.65	2.95
BA StyleIE	3.8	3.9

**Table 9:** AB group survey means.

	Self-Rate Success	Perceived Usefulness
AB DeepIE	2.75	3.15
AB StyleIE	3.7	4.4

**Table 10:** BA group survey means.

	Self-Rate Success	Perceived Usefulness
BA DeepIE	2.65	2.95
BA StyleIE	3.8	3.9

**Table 11:** Aggregated survey means.

	Self-Rate Success	Perceived Usefulness
DeepIE	2.7	3.05
StyleIE	3.75	4.15

**Table 12:** Generation means.

	Total Generations	Generation of Best Image	Ratio
AB DeepIE	12.2	8.8	0.67
AB StyleIE	10.45	7.8	0.73
BA DeepIE	12.68	6.78	0.56
BA StyleIE	13.47	9.89	0.75

## Data Availability

Data is contained within the article or supplementary material.

## Supplementary Materials

The following materials are available online at https://www.mdpi.com/1099-4300/23/1/11/s1: our results and statistical test appendix. The code for experiments is avaiable at https://github.com/cto300/Interactive-StyleGAN.

## Abbreviations

The following abbreviations are used in this manuscript:

IEC	Interactive Evolutionary Computation
EC	Evolutionary Computation
EArt	Evolutionary Art
GAN	Generative Adversarial Network
StyleGAN	Style-Based Generative Adversarial Network
ProGAN	Progressive growing Generative Adversarial Network
DeepIE	Deep Interactive Evolution
StyleIE	Style-Based Deep Interactive Evolution
