# Supplementary material for: Improving Deep Interactive Evolution with a Style-Based Generator for Artistic Expression and Creative Exploration
Source: Entropy (Basel). 2020 Dec 24;23(1):11. doi: 10.3390/e23010011 (PMC7823808; doi:10.3390/e23010011)
Supplement: Supplementary file 1 [file entropy-23-00011-s001.pdf]

entropy

MDPI

Article

Improving Deep Interactive Evolution with Style-Based Generator for Artistic Expression and Creative Exploration - Appendix

Carlos Tejeda Ocampo [1]*, Armando López-Cuevas [2] and Hugo Terashima-Marin [3]

[1] Tecnologico de Monterrey, School of Engineering and Sciences: a01170840@itesm.mx
[1] Tecnologico de Monterrey, School of Engineering and Sciences: acuevas@tec.mx
[3] Tecnologico de Monterrey, School of Engineering and Sciences; terashima@tec.mx
* Correspondence: carlos_tejeda300@hotmail.com

Received: date; Accepted: date; Published: date

Abstract: Deep Interactive Evolution (DeepIE) combines the capacity of Interactive Evolutionary Computation (IEC) to capture user's preference with the domain-specific robustness of a trained Generative Adversarial Network (GAN) generator, allowing the user to control the GAN output through evolutionary exploration of the latent space. However, traditional GAN latent space presents feature entanglement, which limits the practicability of possible applications of DeepIE. In this paper, we implement DeepIE within a Style-Based generator from a Style-GAN model trained on the WikiArt dataset and propose StyleIE, a variation of DeepIE that takes advantage of the secondary disentangled latent space in the Style-Based generator. We performed two AB/BA crossover user tests that compared the performance of DeepIE against StyleIE for art generation. Self-rated evaluation of the performance was collected through a questionnaire. Findings from the tests suggest that StyleIE and DeepIE perform equally in tasks with open-ended goals with relaxed constraints, but StyleIE performs better in more close-ended and constrained tasks.

Keywords: generative adversarial networks; interactive evolutionary computation; deep interactive evolution; StyleGAN; latent space exploration; neural art; evolutionary art

Appendix A Experiment 1

Appendix A.1 Raw survey data

Table A1. Raw Survey Data from AB group in Experiment 1

AB DeepIE Self-Rated Success	Perceived usefulness	AB StyleIE Self-Rated Success	Perceived usefulness	Preference
3	4	2	3	DeepIE
3	5	4	5	StyleIE
4	4	4	4	NA
4	4	4	4	DeepIE
3	3	4	4	StyleIE
4	5	2	2	DeepIE
5	4	4	4	StyleIE
4	4	4	4	DeepIE
4	5	4	5	StyleIE
5	5	3	3	StyleIE

Table A2. Raw Survey Data from BA group in Experiment 1

BA DeepIE Self-Rated Success	Perceived usefulness	BA StyleIE Self-Rated Success	Perceived usefulness	Preference
5	4	5	4	DeepIE
5	4	5	4	DeepIE
3	3	4	4	DeepIE
4	4	4	4	NA
3	1	1	1	NA
3	5	5	5	NA
3	4	1	3	NA
4	4	4	3	DeepIE
5	5	3	3	StyleIE
4	3	5	5	NA

Appendix A.2 Quantitative Survey Analysis

Appendix A.2.1 Self-Rated Success reported in Survey

Table A3. Self-Rated Success reported in Survey

AB DeepIE	*AB StyleIE*	*BA DeepIE*	*BA StyleIE*
3	2	5	5
3	4	5	5
4	4	3	4
4	4	4	4
3	4	3	1
4	2	3	5
5	4	3	1
4	4	4	4
4	4	5	3
5	3	4	5

Table A4. ANOVA for Self-Rated Success

Source of variation	*Square Sum*	*DF*	*Mean squares*	*F*	*P-values*	*F-critical*
Between groups	1.1	3	0.36666	0.32673	0.80600	2.86626
Among groups	40.4	36	1.12222			
Total	41.5	39				

Appendix A.2.2 Perceived usefulness reported in Survey

Table A5. Perceived usefulness reported in Survey

AB DeepIE	*AB StyleIE*	*BA DeepIE*	*AB StyleIE*
4	3	4	4
5	5	4	4
4	4	3	4
4	4	4	4
3	4	1	1
5	2	5	5
4	4	4	3
4	4	4	3
5	5	5	3
5	3	3	5

Table A6. ANOVA for Perceived usefulness

Source of variation	*Square Sum*	*DF*	*Mean squares*	*F*	*P-value*	*F-critical*
Between groups	2.9	3	0.96667	0.96132	0.4215	2.86626
Among group	36.2	36	1.00555			
Total	39.1	39				

Appendix A.3 Generations and ratios

Table A7. Ratio best/total

AB DeepIE	*AB StyleIE*	*BA DeepIE*	*BA StyleIE*
0.94736842	0.5	0.83333333	0.83333333
0.88888889	0.85714286	0.85714286	0.77777778
0.84615385	0.8	0.71428571	0.71428571
0.8125	0.90909091	0.85714286	1
0.14285714	0.92857143	0.66666667	0.88888889
0.85714286	0.83333333	0.66666667	0.75
0.85714286	0.85714286	0.75	0.7
0.85714286	0.77777778	0.77777778	0.57142857
0.875	0.75	1	0.6
0.85714286	0.625	0.71428571	0.66666667

Table A8. ANOVA for Ratio best/total

Source of variation	*Square Sum*	*DF*	*Mean squares*	*F*	*P-values*	*F-critical*
Among groups	0.0109	3	0.0036	0.14765	0.9305	2.8662
Between groups	0.8920	36	0.0247			
Total	0.90298	39				

Table A9. Total Number of generations by method

DeepIE	*StyleIE*
19	20
9	7
13	10
16	11
14	14
7	6
7	7
7	9
8	12
7	8
6	6
9	7
7	7
6	7
9	9
12	6
10	8
7	9
5	8
9	7

Table A10. Paired t-test for total number of generations by method

	DeepIE	*StyleIE*
Mean	9.35	8.9
Variance	13.5026316	11.2526316
Observations	20	20
Pearson Correlation Coefficient	0.75020836	
Hypothetical mean difference	0	
Df	19	
T-Stat	0.80430745	
P(T<=t) one-tail	0.21558438	
t-critical (one.tail)	1.72913281	
P(T<=t) two-tail	0.43116876	
t-critical (two-tail)	2.09302405	

Table A11. Generation of best image found by method

DeepIE	*StyleIE*
18	10
8	6
11	8
13	10
2	13
6	5
6	6
6	7
7	9
6	5
5	5
7	6
5	5
6	6
8	6
9	4
7	6
4	7
3	8
6	5

Table A12. Paired t-test for generation of best image found by method

	DeepIE	*StyleIE*
Mean	7.15	6.85
Variance	12.7657895	4.97631579
Observations	20	20
Pearson Correlation Coefficient	0.19447057	
Hypothetical mean difference	0	
Df	19	
T-Stat	0.35061823	
P(T<=t) one-tail	0.36486607	
t-critical (one-tail)	1.72913281	
P(T<=t) two-tail	0.72973214	
t-critical (two-tail)	2.09302405	

Table A13. Total Number of generations by Group

AB	*BA*
20	6
7	7
10	7
11	7
14	9
6	6
7	8
9	9
12	8
8	7

Table A14. Paired t-test for Number of generations by Group

	AB	*BA*
Mean	10.4	7.4
Variance	17.6	1.15555556
Observations	10	10
Pearson Correlation Coefficient	-0.06405884	
Hypothetical mean difference	0	
Df	9	
T-Stat t	2.15758486	
P(T<=t) one-tail	0.02965007	
t-critical (one-tail)	1.83311293	
P(T<=t) two-tail	0.05930014	
t-critical (two-tail)	2.26215716	

Table A15. Generation of best image found by Group

AB	*BA*
10	5
6	6
8	5
10	6
13	6
5	4
6	6
7	7
9	8
5	5

Table A16. Paired t-test for best image found by Group

	AB	*BA*
Mean	7.9	5.8
Variance	6.76666667	1.28888889
Observations	10	10
Pearson Correlation Coefficient	0.29346544	
Hypothetical mean difference	0	
Df	9	
T-Stat	2.64109739	
P(T<=t) one-tail	0.0134327	
t-critical (one-tail)	1.83311293	
P(T<=t) two-tail	0.0268654	
t-critical (two-tail)	2.26215716	

Appendix A.4 Aggregated surveys

Table A17. Self-Rated Success in both methods

DeepIE	*StyleIE*
3	2
3	4
4	4
4	4
3	4
4	2
5	4
4	4
4	4
5	3
5	5
5	5
3	4
4	4
3	1
3	5
3	1
4	4
5	3
4	5

Table A18. Paired t-test for Self-Rated Success in both methods

	DeepIE	*StyleIE*
Mean	3.9	3.6
Variance	0.621052632	1.51578947
Observations	20	20
Pearson Correlation Coefficient	0.336321353	
Hypothetical mean difference	0	
Df	19	
T-Stat	1.101256536	
P(T<=t) one-tail	0.142270576	
t-critical (one-tail)	1.729132812	
P(T<=t) two-tail	0.284541151	
t-critical (two-tail)	2.093024054	

Table A19. Self-Rated Usefulness of both methods

DeepIE	*StyleIE*
4	3
5	5
4	4
4	4
3	4
5	2
4	4
4	4
5	5
5	3
4	4
4	4
3	4
4	4
1	1
5	5
4	3
4	3
5	3
3	5

Table A20. Paired t-test for Self-Rated Success in both methods

	DeepIE	*StyleIE*
Mean	4	3.7
Variance	0.947368421	1.06315789
Observations	20	20
Pearson Correlation Coefficient	0.367101452	
Hypothetical mean difference	0	
Df	19	
T-Stat	1.188790621	
P(T<=t) one-tail	0.1245842	
t-critical (one-tail)	1.729132812	
P(T<=t) two-tail	0.2491684	
t-critical (two-tail)	2.093024054	

Appendix B Experiment 2

Appendix B.1 Raw survey data

Table A21. Raw Survey Data from AB group in Experiment 2

AB DeepIE Self-Rated Success	Perceived usefulness	AB StyleIE Self-Rated Success	Perceived usefulness	Preference
2	3	4	4	StyleIE
3	3	4	5	StyleIE
3	4	4	4	StyleIE
5	5	4	5	NA
2	3	1	3	DeepIE
1	2	2	3	StyleIE
2	3	2	3	NA
3	5	4	5	NA
4	4	3	4	DeepIE
4	5	5	5	StyleIE
3	5	3	5	StyleIE
3	3	5	5	StyleIE
3	3	4	4	StyleIE
1	1	5	5	StyleIE
2	2	4	4	StyleIE
2	2	5	5	StyleIE
5	4	4	5	StyleIE
4	3	3	4	StyleIE
2	2	4	5	StyleIE
1	1	4	5	StyleIE

Table A22. Raw Survey Data from BA group in Experiment 2

BA StyleIE Self-Rated Success	Perceived usefulness	BA DeepIE Self-Rated Success	Perceived usefulness	Preference
5	5	2	2	StyleIE
4	3	1	1	StyleIE
2	3	4	5	DeepIE
3	3	2	3	StyleIE
4	5	3	3	StyleIE
4	3	4	4	NA
4	4	3	3	StyleIE
4	4	2	2	StyleIE
2	3	3	2	StyleIE
1	2	2	2	NA
4	3	2	3	StyleIE
4	4	3	3	StyleIE
4	4	3	3	StyleIE
4	4	3	3	StyleIE
5	5	2	1	StyleIE
4	4	1	1	StyleIE
5	5	4	5	StyleIE
4	4	4	4	NA
4	5	3	5	DeepIE
5	5	2	4	StyleIE

Appendix B.2 Quantitative Survey Analysis

Appendix B.2.1 ANOVA Survey

Table A23. Self-Rated success in both groups and methods

AB DeepIE	*BA DeepIE*	*BA StyleIE*	*BA StyleIE*
2	2	4	5
3	1	4	4
3	4	4	2
5	2	4	3
2	3	1	4
1	4	2	4
2	3	2	4
3	2	4	4
4	3	3	2
4	2	5	1
3	2	3	4
3	3	5	4
3	3	4	4
1	3	5	4
2	2	4	5
2	1	5	4
5	4	4	5
4	4	3	4
2	3	4	4
1	2	4	5
5	5	2	1
4	4	1	1
5	5	4	5
4	4	4	4
4	5	3	5
5	5	2	4

Table A24. ANOVA for Self-Rated success

Source of variation	*Square Sum*	*DF*	*Mean squares*	*F*	*P-value*	*F-critical*
Between groups	22.25	3	7.4166	6.4272	0.0006	2.7249
Among groups	87.7	76	1.153			
Total	109.95	79				

Table A25. Perceived usefulness in both groups and methods

AB DeepIE	*BA DeepIE*	*BA StyleIE*	*BA StyleIE*
2	3	5	4
1	3	3	5
5	4	3	4
3	5	3	5
3	3	5	3
4	2	3	3
3	3	4	3
2	5	4	5
2	4	3	4
2	5	2	5
3	5	3	5
3	3	4	5
3	3	4	4
3	1	4	5
1	2	5	4
1	2	4	5
5	4	5	5
4	3	4	4
5	2	5	5
4	1	5	5
5	5	2	1
4	4	1	1
5	5	4	5
4	4	4	4
4	5	3	5
5	5	2	4

Table A26. ANOVA for perceived usefulness

ANOVA for perceived usefulness	*Square Sum*	*DF*	*Mean squares*	*F*	*P-value*	*F-critical*
Between groups	27.1	3	9.033	7.79265	0.0001	2.7249
Among groups	88.1	76	1.1592			
Total	115.2	79				

Appendix B.3 Independent t-test on survey

Table A27. Self-Rated success for DeepIE in both groups

AB DeepIE	*BA DeepIE*
2	2
3	1
3	4
5	2
2	3
1	4
2	3
3	2
4	3
4	2
3	2
3	3
3	3
1	3
2	2
2	1
5	4
4	4
2	3
1	2

Table A28. F-test for Self-Rated Success for DeepIE in both groups

	AB DeepIE	*BA DeepIE*
Mean	2.75	2.65
Variance	1.46052632	0.87105263
Observations	20	20
DF	19	19
F	1.67673716	
P(F<=f) one-tail	0.13442467	
F-critical (one-tail	2.1682516	

Table A29. Independent t-test for Self-Rated Success in DeepIE both groups assuming equal variances

	AB DeepIE	*BA DeepIE*
Mean	2.75	2.65
Variance	1.46052632	0.87105263
Observations	20	20
Pooled Variance	1.16578947	
Hypothetical mean differences	0	
DF	38	
T-stat	0.29288015	
P(T<=t) one-tail	0.38560359	
t-critical (one-tail)	1.68595446	
P(T<=t) two-tail	0.77120718	
t-critical (two-tail)	2.02439416	

Table A30. Perceived usefulness for DeepIE in both groups

AB DeepIE	*BA DeepIE*
3	2
3	1
4	5
5	3
3	3
2	4
3	3
5	2
4	2
5	2
5	3
3	3
3	3
1	3
2	1
2	1
4	5
3	4
2	5
1	4

Table A31. F-test for perceived usefulness for DeepIE in both groups

	AB DeepIE	*BA DeepIE*
Mean	3.15	2.95
Variance	1.60789474	1.62894737
Observations	20	20
DF	19	19
F	0.98707593	
P(F<=f) one-tail	0.48883888	
F-critical (one-tail)	0.46120109	

Table A32. Independent t-test for perceived usefulness in DeepIE for both groups assuming equal variances

	AB DeepIE	*BA DeepIE*
Mean	3.15	2.95
Variance	1.60789474	1.62894737
Observations	20	20
Pooled Variance	1.61842105	
Hypothetical mean differences	0	
DF	38	
T-stat	0.49714633	
P(T<=t) one-tail	0.31097511	
t-critical (one-tail)	1.68595446	
P(T<=t) two-tail	0.62195022	
t-critical (two-tail)	2.02439416	

Table A33. Self-Rated Success for StyleIE in both groups

AB StyleIE	*BA StyleIE*
4	5
4	4
4	2
4	3
1	4
2	4
2	4
4	4
3	2
5	1
3	4
5	4
4	4
5	4
4	5
5	4
4	5
3	4
4	4
4	5

Table A34. F-test for Self-Rated Success for StyleIE Success in both groups

	AB StyleIE	*BA StyleIE*
Mean	4.4	3.9
Variance	0.56842105	0.83157895
OBservations	20	20
DF	19	19
F	0.6835443	
P(F<=f) one-tail	0.20726366	
F-critical (one-tail)	0.46120109	

Table A35. Independent t-test for Self-Rated Success in StyleIE for both groups assuming equal variances

	AB StyleIE	*BA StyleIE*
Mean	3.7	3.8
Variance	1.16842105	1.11578947
Observations	20	20
Pooled variance	1.14210526	
Hypothetical mean differences	0	
DF	38	
T-stat	-0.29590134	
P(T<=t) one-tail	0.38445824	
t-critical (one-tail)	1.68595446	
P(T<=t) two-tail	0.76891648	
t-critical (two-tail)	2.02439416	

Table A36. Perceived usefulness for StyleIE in both groups

AB StyleIE	*BA StyleIE*
4	5
5	3
4	3
5	3
3	5
3	3
3	4
5	4
4	3
5	2
5	3
5	4
4	4
5	4
4	5
5	4
5	5
4	4
5	5
5	5

	AB StyleIE	*BA StyleIE*
Mean	4.4	3.9
Variance	0.56842105	0.83157895
Observations	20	20
DF	19	19
F	0.6835443	
P(F<=f) one-tail	0.20726366	
F-critical (one-tail)	0.46120109	

	AB StyleIE	*BA StyleIE*
Mean	4.4	3.9
Variance	0.56842105	0.83157895
Observations	20	20
Pooled Variance	0.7	
Hypothetical mean differences	0	
DF	38	
T-stat	1.88982237	
P(T<=t) one-tail	0.03321409	
t-critical (one-tail)	1.68595446	
P(T<=t) two-tail	0.06642817	
t-critical (two-tail)	2.02439416	

Appendix B.4 Paired t-test on survey

Table A37. Self-Rated success DeepIE vs StyleIE in AB

AB DeepIE	*AB StyleIE*
2	4
3	4
3	4
5	4
2	1
1	2
2	2
3	4
4	3
4	5
3	3
3	5
3	4
1	5
2	4
2	5
5	4
4	3
2	4
1	4

Table A38. Paired t-test Self-Rated Success DeepIE vs StyleIE in AB

	AB DeepIE	*AB StyleIE*
Mean	2.75	3.7
Variance	1.46052632	1.16842105
Observation	20	20
Pearson correlation coefficient	0.1410133	
Hypothetical mean differences	0	
DF	19	
t-stat	-2.82575054	
P(T<=t) one-tail	0.0053999	
t-critical (one-tail)	1.72913281	
P(T<=t) two-tail	0.01079979	
t-critical (two-tail)	2.09302405	

Table A39. Perceived usefulness DeepIE vs StyleIE in AB

DeepIE	*StyleIE*
3	4
3	5
4	4
5	5
3	3
2	3
3	3
5	5
4	4
5	5
5	5
3	5
3	4
1	5
2	4
2	5
4	5
3	4
2	5
1	5

Table A40. Paired t-test perceived usefulness DeepIE vs StyleIE in AB

	DeepIE	*StyleIE*
Mean	3.15	4.4
Variance	1.60789474	0.56842105
Observations	20	20
Pearson correlation coefficient	0.15414894	
Hypothetical mean differences	0	
DF	19	
t-stat	-4.07533945	
P(T<=t) one-tail	0.00032253	
t-critical (one-tail)	1.72913281	
P(T<=t) two-tail	0.00064507	
t-critical (two-tail)	2.09302405	

Table A41. Self-Rated success DeepIE vs StyleIE in BA

DeepIE	*StyleIE*
2	5
1	4
4	2
2	3
3	4
4	4
3	4
2	4
3	2
2	1
2	4
3	4
3	4
3	4
2	5
1	4
4	5
4	4
3	4
2	5

Table A42. Paired t-test Self-Rated success DeepIE vs StyleIE in BA

	DeepIE	*StyleIE*
Mean	2.65	3.8
Variance	0.87105263	1.11578947
Observations	20	20
Pearson correlation coefficient	-0.07474138	
Hypothetical mean differences	0	
DF	19	
t-stat	-3.52041781	
P(T<=t) one-tail	0.00114343	
t-critical (one-tail)	1.72913281	
P(T<=t) two-tail	0.00228686	
t-critical (two-tail)	2.09302405	

Table A43. Perceived usefulness DeepIE vs StyleIE in BA

DeepIE	*StyleIE*
2	5
1	3
5	3
3	3
3	5
4	3
3	4
2	4
2	3
2	2
3	3
3	4
3	4
3	4
1	5
1	4
5	5
4	4
5	5
4	5

Table A44. Paired t-test perceived usefulness DeepIE vs StyleIE in BA

	DeepIE	*StyleIE*
Mean	2.95	3.9
Variance	1.62894737	0.83157895
Observations	20	20
Pearson correlation coefficient	0.17636229	
Hypothetical mean differences	0	
DF	19	
t-statt	-2.96730148	
P(T<=t) one-tail	0.0039569	
t-critical (one-tail)	1.72913281	
P(T<=t) two-tail	0.0079138	
t-critical (two-tail)	2.09302405	

Appendix B.5 Aggregated results

Table A45. Aggregated Self-Rated success DeepIE vs StyleIE

DeepIE	*StyleIE*
2	4
3	4
3	4
5	4
2	1
1	2
2	2
3	4
4	3
4	5
3	3
3	5
3	4
1	5
2	4
2	5
5	4
4	3
2	4
1	4
2	5
1	4
4	2
2	3
3	4
4	4
3	4
2	4
3	2
2	1
2	4
3	4
3	4
3	4
2	5
1	4
4	5
4	4
3	4
2	5

Table A46. Paired t-test aggregated Self-Rated Success DeepIE vs StyleIE

	DeepIE	*StyleIE*
Mean	2.7	3.75
Variance	1.13846154	1.11538462
Observations	40	40
Pearson correlation coefficient	0.04550864	
Hypothetical mean differences	0	
DF	39	
t-stat	-4.52762676	
P(T<=t) one-tail	2.7444E-05	
t-critical (one-tail)	1.68487512	
P(T<=t) two-tail	5.4887E-05	
t-critical (two-tail)	2.02269092	

Table A47. Perceived usefulness DeepIE vs StyleIE in BA

DeepIE	*StyleIE*
2	5
1	3
5	3
3	3
3	5
4	3
3	4
2	4
2	3
2	2
3	3
3	4
3	4
3	4
1	5
1	4
5	5
4	4
5	5
4	5
3	4
3	5
4	4
5	5
3	3
2	3
3	3
5	5
4	4
5	5
5	5
3	5
3	4
1	5
2	4
2	5
4	5
3	4
2	5
1	5

Table A48. Paired t-test perceived usefulness DeepIE vs StyleIE

	DeepIE	*StyleIE*
Mean	3.05	4.15
Variance	1.58717949	0.74615385
Observations	40	40
Pearson correlation coefficient	0.18142574	
Hypothetical mean differences	0	
DF	39	
t-stat	-4.99682439	
P(T<=t) one-tail	6.3252E-06	
t-critical (one-tail)	1.68487512	
P(T<=t) two-tail	1.265E-05	
(two-tail)	2.02269092	

Appendix B.6 Generations and ratios

Table A49. Ratio best image generation / total generation

AB DeepIE	*AB StyleIE*	*BA DeepIE*	*BA Style*
0.94117647	0.75	0.4	0.875
0.8125	0.88888889	0.6	0.9
0.95	0.875	0.35	0.85714286
0.66666667	0.85714286	0.75	0.875
0.27272727	0.75	0.45	0.52631579
0.88235294	0.68421053	0.4	0.90909091
0.65	0.4	0.33333333	0.83333333
0.64285714	0.86666667	0.25	0.45
0.95	0.75	0.9	0.9375
0.42857143	0.71428571	0.91666667	0.88888889
0.46666667	0.7	0.8	0.77777778
0.83333333	0.9	0.88888889	0.81818182
0.22222222	0.8	0.72727273	0.63636364
0.375	0.77777778	0.92307692	0.9
0.85714286	0.75	0.14285714	0.7
0.85714286	0.71428571	0.55555556	0.75
0.91666667	0.375	0.75	0.625
0.71428571	0.88888889	0.15	0.44444444
0.33333333	0.7	0.53846154	0.68421053
0.76923077	0.625	0.4	0.72727273

Table A50. ANOVA for Ratio best/total

Source of vatiarion	*Square sum*	*DF*	*Square mean*	*F*	*P-value*	*F-critical*
Between groups	0.4641	3	0.1547	3.6397	0.0164	2.7249
Among groups	3.230	76	0.04250			
Total	3.69435862	79				

Table A51. Total generations in AB

DeepIE	*StyleIE*
10	8
20	20
20	14
8	8
20	19
15	11
6	6
20	20
10	16
12	9
10	18
9	11
11	11
13	10
7	10
9	20
8	8
20	18
13	19
15	11

Table A52. Paired t-test for total generations in AB

	DeepIE	*StyleIE*
Mean	12.8	13.35
Variance	23.7473684	23.7131579
Observations	20	20
Pearson correlation coefficient	0.60194083	
Hypothetical mean differences	0	
DF	19	
t-stat	-0.56589715	
P(T<=t) one-tail	0.28904335	
t-critical (one-tail)	1.72913281	
P(T<=t) two-tail	0.57808669	
t-critical (two-tail)	2.09302405	

Table A53. Generation of best image in AB

4	7
12	18
7	12
6	7
9	10
6	10
2	5
5	9
9	15
11	8
8	14
8	9
8	7
12	9
1	7
5	15
6	5
3	8
7	13
6	8

Table A54. Paired t-test for generation of best image in AB

	DeepIE	*StyleIE*
Mean	6.75	9.8
Variance	9.14473684	12.8
Observations	20	20
Pearson correlation coefficient	0.47674032	
Hypothetical mean differences	0	
DF	19	
t-stat	-3.99985856	
P(T<=t) one-tail	0.00038322	
t-critical (one-tail)	1.72913281	
P(T<=t) two-tail	0.00076644	
t-critical (two-tail)	2.09302405	

Table A55. Generation ratios in AB

0.4	0.875
0.6	0.9
0.35	0.85714286
0.75	0.875
0.45	0.52631579
0.4	0.90909091
0.33333333	0.83333333
0.25	0.45
0.9	0.9375
0.91666667	0.88888889
0.8	0.77777778
0.88888889	0.81818182
0.72727273	0.63636364
0.92307692	0.9
0.14285714	0.7
0.55555556	0.75
0.75	0.625
0.15	0.44444444
0.53846154	0.68421053
0.4	0.72727273

Table A56. Paired t-test for ratio in AB

	DeepIE	*StyleIE*
Mean	0.67709382	0.73835735
Variance	0.05913607	0.02068346
Observations	20	20
Pearson correlation coefficient	-0.04506963	
Hypothetical mean differences	0	
DF	19	
t-stat	-0.95115487	
P(T<=t) one-tail	0.17673475	
t-critical (one-tail)	1.72913281	
P(T<=t) two-tail	0.35346949	
t-critical (two-tail)	2.09302405	

Table A58. Paired t-test for Total generations in AB

	DeepIE	*StyleIE*
Mean	12.8	13.35
Variance	23.7473684	23.7131579
Observations	20	20
Pearson correlation coefficient	0.60194083	
Hypothetical mean differences	0	
DF	19	
t-stat	-0.56589715	
P(T<=t) one-tail	0.28904335	
t-critical (one-tail)	1.72913281	
P(T<=t) two-tail	0.57808669	
t-critical (two-tail)	2.09302405	

Table A57. Total generations in BA

DeepIE	*StyleIE*
10	8
20	20
20	14
8	8
20	19
15	11
6	6
20	20
10	16
12	9
10	18
9	11
11	11
13	10
7	10
9	20
8	8
20	18
13	19
15	11

Table A59. Best image generation in BA

DeepIE	*StylIE*
4	7
12	18
7	12
6	7
9	10
6	10
2	5
5	9
9	15
11	8
8	14
8	9
8	7
12	9
1	7
5	15
6	5
3	8
7	13
6	8

Table A60. Paired t-test for generation of best image in BA

	DeepIE	*StyleIE*
Mean	6.75	9.8
Variance	9.14473684	12.8
Observations	20	20
Pearson correlation coefficient	0.47674032	
Hypothetical mean differences	0	
DF	19	
t-stat	-3.99985856	
P(T<=t) one-tail	0.00038322	
t-critical (one-tail)	1.72913281	
P(T<=t) two-tail	0.00076644	
t-critical (two-tail)	2.09302405	

Table A61. Generation ratios in BA

DeepIE	*StyleIE*
0.4	0.875
0.6	0.9
0.35	0.85714286
0.75	0.875
0.45	0.52631579
0.4	0.90909091
0.33333333	0.83333333
0.25	0.45
0.9	0.9375
0.91666667	0.88888889
0.8	0.77777778
0.88888889	0.81818182
0.72727273	0.63636364
0.92307692	0.9
0.14285714	0.7
0.55555556	0.75
0.75	0.625
0.15	0.44444444
0.53846154	0.68421053
0.4	0.72727273

Table A62. Paired t-test for ratio in BA

	DeepIE	*StyleIE*
Mean	0.56130564	0.75577614
Variance	0.0665905	0.02360343
Observations	20	20
Pearson correlation coefficient	0.48539476	
Hypothetical mean differences	0	
DF	19	
t-stat	-3.82468868	
P(T<=t) one-tail	0.00057174	
t-critical (one-tail)	1.72913281	
P(T<=t) two-tail	0.00114348	
t-critical (two-tail)	2.09302405	

Table A63. Aggregated Total generations

DeepIE	*StyleIE*
17	20
16	18
20	8
6	7
11	8
17	19
20	10
14	15
20	8
7	7
15	10
12	10
9	10
8	9
7	8
7	7
12	8
7	9
6	10
13	8
10	8
20	20
20	14
8	8
20	19
15	11
6	6
20	20
10	16
12	9
10	18
9	11
11	11
13	10
7	10
9	20
8	8
20	18
13	19
15	11

Table A64. Paired t test for aggregated Total generations

	DeepIE	*StyleIE*
Mean	12.5	11.9
Variance	23.6923077	21.8871795
Observations	40	40
Pearson correlation coefficient	0.51683212	
Hypothetical mean differences	0	
DF	39	
t-stat	0.80828654	
P(T<=t) one-tail	0.21191443	
t-critical (one-tail)	1.68487512	
P(T<=t) two-tail	0.42382886	
t-critical (two-tail)	2.02269092	

Table A65. Aggregated best image generation

DeepIE	*StyleIE*
16	15
13	16
19	7
4	6
3	6
15	13
13	4
9	13
19	6
3	5
7	7
10	9
2	8
3	7
6	6
6	5
11	3
5	8
2	7
10	5
4	7
12	18
7	12
6	7
9	10
6	10
2	5
5	9
9	15
11	8
8	14
8	9
8	7
12	9
1	7
5	15
6	5
3	8
7	13
6	8

Table A66. Paired t test for aggregated best image generation

	DeepIE	*StyleIE*
Mean	7.775	8.8
Variance	20.6916667	13.7025641
Observations	40	40
Pearson correlation coefficient	0.27288191	
Hypothetical mean differences	0	
DF	39	
t-stat	-1.29126366	
P(T<=t) one-tail	0.1021068	
t-critical (one-tail)	1.68487512	
P(T<=t) two-tail	0.2042136	
t-critical (two-tail)	2.02269092	

Table A67. Aggregated generation ratios

DeepIE	*StyleI*
0.94117647	0.75
0.8125	0.88888889
0.95	0.875
0.66666667	0.85714286
0.27272727	0.75
0.88235294	0.68421053
0.65	0.4
0.64285714	0.86666667
0.95	0.75
0.42857143	0.71428571
0.46666667	0.7
0.83333333	0.9
0.22222222	0.8
0.375	0.77777778
0.85714286	0.75
0.85714286	0.71428571
0.91666667	0.375
0.71428571	0.88888889
0.33333333	0.7
0.76923077	0.625
0.4	0.875
0.6	0.9
0.35	0.85714286
0.75	0.875
0.45	0.52631579
0.4	0.90909091
0.33333333	0.83333333
0.25	0.45
0.9	0.9375
0.91666667	0.88888889
0.8	0.77777778
0.88888889	0.81818182
0.72727273	0.63636364
0.92307692	0.9
0.14285714	0.7
0.55555556	0.75
0.75	0.625
0.15	0.44444444
0.53846154	0.68421053
0.4	0.72727273

Table A68. Paired t test for aggregated ratios

	DeepIE	*StyleIE*
Mean	0.61919973	0.74706674
Variance	0.06468908	0.02165347
Observations	40	40
Pearson correlation coefficient	0.21615913	
Hypothetical mean differences	0	
DF	39	
t-stat	-3.05306715	
P(T<=t) one-tail	0.00203367	
t-critical (one-tail)	1.68487512	
P(T<=t) two-tail	0.00406733	
t-critical (two-tail)	2.02269092	

© 2020 by the authors. Licensee MDPI, Basel, Switzerland. This article is an open access article distributed under the terms and conditions of the Creative Commons Attribution (CC BY) license (http://creativecommons.org/licenses/by/4.0/).
